# Loss of HtrA1 serine protease induces synthetic modulation of aortic vascular smooth muscle cells

**DOI:** 10.1371/journal.pone.0196628

**Published:** 2018-05-16

**Authors:** Muthi Ikawati, Masashi Kawaichi, Chio Oka

**Affiliations:** 1 Laboratory of Gene Function in Animals, Nara Institute of Science and Technology, Takayama, Ikoma, Nara, Japan; 2 Department of Pharmaceutical Chemistry, Faculty of Pharmacy, Universitas Gadjah Mada, Sekip Utara, Yogyakarta, Indonesia; Max Delbruck Centrum fur Molekulare Medizin Berlin Buch, GERMANY

## Abstract

Homozygous mutations of human *HTRA1* cause cerebral autosomal recessive arteriopathy with subcortical infarcts and leukoencephalopathy (CARASIL). *HtrA1*^*-/-*^ mice were examined for arterial abnormalities. Although their cerebral arteries were normal, the thoracic aorta was affected in *HtrA1*^*-/-*^ mice. The number of vascular smooth muscle cells (VSMCs) in the aorta was increased in *HtrA1*^*-/-*^ mice of 40 weeks or younger, but decreased thereafter. The cross-sectional area of the aorta was increased in *HtrA1*^*-/-*^ mice of 40 weeks or older. Aortic VSMCs isolated from *HtrA1*^*-/-*^ mice rapidly proliferated and migrated, produced high MMP9 activity, and were prone to oxidative stress-induced cell death. *HtrA1*^*-/-*^ VSMCs expressed less smooth muscle α-actin, and more vimentin and osteopontin, and responded to PDGF-BB more strongly than wild type VSMCs, indicating that *HtrA1*^*-/-*^ VSMCs were in the synthetic phenotype. The elastic lamina was disrupted, and collagens were decreased in the aortic media. Calponin in the media was decreased, whereas vimentin and osteopontin were increased, suggesting a synthetic shift of VSMCs in vivo. Loss of *HtrA1* therefore skews VSMCs toward the synthetic phenotype, induces MMP9 expression, and expedites cell death. We propose that the synthetic modulation is the primary event that leads to the vascular abnormalities caused by *HtrA1* deficiency.

## Introduction

HtrA is a family of serine proteases that is highly conserved among species from bacteria to plants and humans [1]. A major common function of HtrA family members is in protein quality control under various stress conditions in various cellular compartments [2]. DegP, for example, a bacterial HtrA protease, recognizes misfolded proteins in the periplasm and digests them at high temperatures, or re-folds them with its chaperone activity at low temperatures [3–5]. Expression of DegP is induced by stressors such as heat [4, 6], ethanol treatment [7], and oxidative stress [8]. Mammalian HtrA2 is essential for mitochondrial functions and is thought to be involved in protein quality control in the intermembrane space [9].

Functions of mammalian secretory HtrAs (HtrA1, 3, and 4) are largely unknown. HtrA1 exhibits two activities: it degrades various substrates including extracellular matrix (ECM) proteins, and it inhibits the signaling of transforming growth factor (TGF)-β [10, 11]. Contradictory data have also been reported, namely that HtrA1 facilitates TGF-β signaling [12]. HtrA1 is implicated in a wide range of human diseases such as arthritis [13, 14], age-related macular degeneration [15–17], cancer [18], and preeclampsia [19, 20]. HtrA1 is overexpressed in arthritic cartilage, and probably contributes to the degradation of cartilage matrix. It may also aggravate arthritis by inhibiting TGF-β, which is essential to maintain healthy cartilage [11]. HtrA1 may be a tumor suppressor: it is down-regulated upon malignant transformation and metastasis, and its overexpression in cancerous cells inhibits their proliferation and migration [18, 21, 22]. HtrA1 is a stress-responsive factor. HtrA1 is induced by oxidative stress and protects cells from oxidation-induced cell death at the expense of promoting cell senescence in retinal pigment epithelial cells [23], a mechanism that may link HtrA1 with the onset of age-related macular degeneration.

Homozygous loss-of-function mutations of human *HTRA1* cause a hereditary cerebral small vessel disease (SVD) called cerebral autosomal recessive arteriopathy with subcortical infarcts and leukoencephalopathy (CARASIL) [24]. Major signs of CARASIL are alopecia, spondylosis, and early-onset dementia that is caused by nonhypertensive cerebral small vessel arteriopathy [25]. Cerebral arteries of CARASIL patients show fibrous intimal proliferation, thickening and splitting of the internal elastic lamina, massive hyaline degeneration of the media, and extensive loss of vascular smooth muscle cells (VSMCs). These damages eventually result in concentric narrowing of the lumen [24, 26–28]. The extensive VSMC loss and reduction in ECM proteins such as fibrillar collagens and fibronectin seem to be the primary abnormalities in CARASIL [28]. Small arteries in tissues other than the brain are also affected mildly [26, 29]. Heterozygous mutations of *HTRA1* were recently reported in a late-onset familial SVD group [30]. It is not known, however, if mice deficient in *HtrA1* show cerebral SVD.

Vascular diseases are frequently accompanied by a phenotypic shift of VSMCs. There are two types of VSMCs, one for contraction and the other for ECM synthesis [31]. Each of these types represents a phenotypic extreme, and in actual blood vessels, VSMCs display a diffuse spectrum of diversity between contractile and synthetic phenotypes. Fully differentiated contractile VSMCs can be de-differentiated into synthetic cells and vice versa, a process called phenotypic switching or phenotypic modulation, which is induced by humoral factors, cell-ECM or cell-cell interactions, mechanical forces, atherogenic stimuli, and various stress conditions. Each phenotype is characterized by distinct cell morphology, proliferation and migration rates, and expression of marker proteins [32, 33]. Matrix metalloproteinases (MMPs) degrade and remodel the ECM in the blood vessel wall, and thereby control cell-ECM or cell-cell contacts, thus playing a key regulatory role in the phenotypic modulation of VSMCs.

We have produced the *HrA1*^*-/-*^ mouse [11, 34] and here examine whether the *HtrA1*^*-/-*^ mouse is valuable as a model for human CARASIL. This study highlights the phenotypic modulation of VSMCs as an initial event caused by *HtrA1* deficiency. The aorta of the *HrA1*^*-/-*^ mouse shows loss of VSMCs as the age progresses. We reveal that the aortic VSMCs of *HtrA1*^*-/-*^ mice are heavily skewed towards the synthetic phenotype with robust cell proliferation and migration, express high MMP9 activity, and are prone to stress-induced cell death.

## Materials and methods

### Mice

*HtrA1*^*-/-*^ mice were generated by standard homologous recombination procedures as previously described [11, 34]. Original 129/B6 *HtrA1*^*-/-*^ mice were backcrossed more than 10 times with BALB/c mice. *HtrA1*^*-/-*^ mice and wild type (WT) mice with BALB/c background were used unless otherwise indicated. Mice were bred in specific pathogen-free conditions according to the standard protocol of the animal facility of Nara Institute of Science and Technology and were sacrificed by an overdose intraperitoneal injection of sodium pentobarbital. This study was approved by the animal welfare sub-committee of Nara Institute of Science and Technology.

### Cell isolation and culture

VSMCs were isolated from cultured explants of aortas from 10-week-old mice as previously described [35] with some modifications [http://dx.doi.org/10.17504/protocols.io.nwydffw]. Medial explants of entire descending thoracic aortas from 2–3 mice were placed in 3.5 cm dishes and cultured in Dulbecco’s modified Eagle’s medium/Ham’s F12 (DMEM/F12) (Gibco) supplemented with 20% fetal bovine serum (FBS) (Sigma) and antibiotics (70 μg/ml penicillin and 50 μg/ml streptomycin). Cells that migrated from the explant were harvested and expanded by culturing in 10% FBS/DMEM/F12 (complete medium). Isolated VSMCs retained HrA1 expression comparable to that in the aorta, at least up to passage 13. VSMCs from passage 7 to 14 were used for the experiments. Three batches each of *HtrA1*^*-/-*^ and WT VSMCs were obtained and most of the experiments were conducted for all batches. For treatment of cells with growth factors or inhibitors, VSMCs were plated into 6-well plates in DMEM/F12 containing 10% FBS and grown to confluence. The cells were then serum-starved for 24 h in 0.1% or 0.5% FBS/DMEM/F12 (starvation medium). Growth factors and inhibitors were then added to the culture at the concentrations listed in S1 Table.

### Cell proliferation

VSMCs (2x10^3^ cells/well) were plated in 96-well plates and cultured in complete medium for 15 h. The medium was then replaced with either a medium containing a different concentration of FBS or one containing 0.5% FBS and either PDGF-BB or IGF-1. Cell numbers were assessed with a Cell Counting Kit-8 (Dojindo Molecular Technologies) according to the manufacturer’s instruction at the indicated time after medium replacement. The absorbance at 450 nm, which represents the living cell number, was normalized to the value for 0 h (time of medium replacement) to calculate fold proliferation. Data are presented as the mean of three or four measurements per condition per experiment.

### Migration assay

Wound-healing assays were performed using an Ibidi culture-insert system [36]. Briefly, 70 μl of cell suspension (1.4x10^4^ cells) was loaded into each well of the culture-insert. After 20 h to allow the cells to attach to the dish, mitomycin C solution (Nacalai Tesque) was added to 1 μg/ml to inhibit cell proliferation. Four hours later, the culture insert was gently removed using tweezers to generate a 500 μm wide cell-free gap. The dish was then washed with PBS to remove unattached cells and filled with medium containing a different concentration of FBS, or with an FBS-free medium supplemented with either PDGF-BB, IGF-1, or inhibitors. The experiments were conducted in triplicate. Images were captured at the indicated time points using an inverted microscope (Nikon Diaphot 300) equipped with a digital camera (Sony NEX-3N).

A migration assay using a modified Boyden chamber method was carried out with Transwell Permeable Support Inserts (Corning) according to the manufacturer’s protocol. The insert and the receiver chambers were rehydrated with pre-warmed starvation medium containing 0.5% FBS, and the cell suspension (1x10^5^ cells in 100 μl) was then loaded into the insert. Five hundred microliters of complete medium, or starvation medium containing PDGF-BB or IGF-1, were added to the receiver chamber. After 30 min to allow the cells to attach, the chambers were assembled. After 24 h, the cells that had migrated into the bottom side of the insert chamber were stained with DAPI. The number of migrated cells was counted in five fields for each insert. Three wells were used per condition per experiment.

### Gelatin zymography

MMP activities in the VSMC culture media were assayed by gelatin zymography as previously described [20]. The culture medium was centrifuged to remove cell debris. The volume of the cleared culture supernatant applied to the zymography gel was adjusted according to the tubulin content in the cell lysate recovered from the same culture, to compensate for differences in cell numbers in each culture. The culture supernatant was mixed with zymograph sample buffer (62 mM Tris-HCl pH 6.8, 2% SDS, 10% glycerol and 0.01% bromophenol blue) and loaded into a well of an 8% SDS-polyacrylamide gel containing 1% gelatin. After electrophoresis and washing the gel in 2.5% (w/v) Triton X-100, 50 mM Tris-HCl pH 7.5, three times each for 20 min, the gel was incubated in a gel development buffer (10 mM CaCl_2_, 150 mM NaCl, 50 mM Tris-HCl pH 7.5, 0.02% NaN_3_) for 48 h at 37°C. Next, the gel was stained with 0.125% Coomassie Brilliant Blue R-250 for 1 h at room temperature and destained in methanol:acetic acid:water (1:2:17). The destained gel was scanned with a CanoScan LiDE 200 (Canon), and the intensities of white bands indicating gelatinolytic activity were measured by ImageJ software (NIH).

### qRT-PCR

Total RNA from VCMCs was extracted using Sepasol-RNA I Super G (Nacalai Tesque) according to the manufacturer’s instructions. After contaminating genomic DNA was degraded with gDNA Eraser, 1 μg of total RNA was reverse transcribed using the PrimeScript RT reagent kit with gDNA Eraser (Takara). cDNA samples were amplified in triplicate using the SYBR qPCR mix (Toyobo) with the LightCycler 96 PCR System (Roche Applied Science). The following conditions were used: denaturation at 95°C for 1 min; 45 cycles of PCR at 95°C for 10 s, 55°C for 30 s, 72°C for 20 s; and final steps at 95°C for 15 s, 60°C for 30 s, and 95°C for 15 s for dissociation curve analysis. A dissocation curve for each PCR product was determined following the LightCycler’s instructions to ensure specific amplification of the target gene. The data were analyzed by the ΔΔtC method, using GAPDH as the internal control. The primers used are listed in S2 Table.

### Cell viability and apoptosis assay

VSMCs (2x10^3^ cells/well) were plated in 96-well plates and cultured in complete medium for 24 h, and then serum-starved for 24 h in DMEM/F12 containing 0.1% FBS. The cells were treated with 0.1 mM or 0.3 mM H_2_O_2_ in the latter medium for 24 h and the cell number was examined using Cell Count Reagent SF (Nacalai Tesque). For the apoptosis assay, VSMCs were grown to confluence on cover glass in 6-well plates. VSMCs were serum-starved as above and then treated with 0.1 or 0.3 mM H_2_O_2_ for 6 h in the starvation medium. Cells were triply stained with an Apoptotic/Necrotic/Healthy Cells Detection Kit (PromoKine) according to the manufacturer’s instruction, or immunostained with anti-cleaved caspase-3 (Asp175) (5A1E) rabbit monoclonal antibody (1:400; catalog #9664; Cell Signaling Technology). The cells were cultured in 6 cm plates, and similarly treated with 0.3 mM H_2_O_2_ in medium containing 0.1% FBS. Cell lysates were then prepared at several time points to assay cleaved caspase-3 by Western blot using the same antibody (1:1,000).

### Histology, histomorphometry, and immunostaining

Mouse tissues were removed and fixed overnight in 4% paraformaldehyde in phosphate-buffered saline (PBS) at 4°C, and then processed for paraffin embedding [http://dx.doi.org/10.17504/protocols.io.nw5dfg6]. The upper and lower half of the descending thoracic aorta was used for histological analyses and tissue extract preparation, respectively. Serial sections (5 μm) were cut from paraffin-embedded tissues, and then stained with hematoxylin and eosin (HE), the elastica van Gieson (EVG) reagent for elastic fibers, and picrosirius red for collagens. To observe myelin, brain sections were stained with luxol fast blue and cresyl violet (Klüver-Barrera staining).

Images of at least three EVG-stained serial sections cut at 100 μm intervals from each aorta were captured for histomorphometry with a light microscope (Olympus BX50) equipped with a digital camera (Nikon DS-Fi1). Aortas from four to ten mice were used for each time point. External and internal circumferences of the media were defined and their lengths were measured using NIS Element Basic Research (Nikon). Histomorphometric parameters of the aorta were defined according to Hart *et al* [37] and Vaja *et al* [38] with modifications as follows: cross-sectional area representing aorta size was the area within the external circumference. Media area was calculated by subtracting the area within the internal circumference from the cross-sectional area. Media thickness was calculated by dividing the media area by the internal circumference. Lumen diameter was determined as the internal circumference divided by π.

Thickening of media was analyzed with EVG-stained sections. Disruption of elastic fibers was analyzed with autofluorescence of HE-stained sections that were observed with a fluorescence microscope (Olympus BX50 microscope fitted with a Nikon DS-2MBWc camera). The aorta was categorized as having uneven thickening if its thickest area was 2.5 times or more as thick as the thinnest area. To assess the number of VSMCs, VSMC nuclei in the aortic media of HE-stained sections were manually counted.

Immunohistochemistry with anti-HtrA1 or anti-HtrA3 serum was carried out using the avidin-biotin complex method as described previously [20]. After DAPI counterstaining, the sections were mounted with an aqueous mounting medium (Fluoromount, Sigma). The same procedures were used to detect VSMC markers. The primary antibodies were diluted in PBS-T (PBS containing 0.25% (w/v) Triton X-100) containing 1% bovine serum albumin. After counterstaining with hematoxylin, the sections were dehydrated in graded ethanol, cleared in xylene, and mounted (Softmount, Wako). Negative controls were obtained by omitting the primary antibody. The antibodies used are listed in S3 Table.

### Western blotting

The lower halves of the descending thoracic aortas were frozen over liquid nitrogen after removal of fat layers, and then thawed and minced on ice [http://dx.doi.org/10.17504/protocols.io.nw9dfh6]. Minced aortas from 3–4 mice were pooled and subjected to extraction. Minced aortas or harvested culture cells were homogenized in a lysis buffer [12] and the lysates were centrifuged [http://dx.doi.org/10.17504/protocols.io.nxadfie]. Protein concentrations of the cleared lysates were determined using Pierce 660 nm Protein Assay Reagent (Thermo Scientific). Equal amounts of proteins or 20 μl of a conditioned medium (for HtrA1 immunoblot) were resolved on a 10% SDS-polyacrylamide gel and then electrotransferred to a PVDF membrane as previously described [23]. The membrane was blocked with TBS-T (Tris-buffered saline containing 0.1% (w/v) Tween-20) containing 0.3% or 5% nonfat dry milk for 1 h at room temperature, and then incubated overnight at 4°C with a diluted primary antibody. The antibodies used and their dilutions are listed in S4 Table. The membrane was then incubated with the appropriate HRP-conjugated secondary antibody (1:5,000; catalog number NA9310; or 1:25,000; catalog number NA9340; Amersham Bioscience). Signals from HRP on the membrane were developed by ECL Prime (Amersham Bioscience) and detected with a Luminescent Image Analyzer LAS4000 (Fujifilm). The band intensities were measured by ImageJ software and normalized with tubulin to calculate relative expression levels.

### Statistical analysis

Data are presented as mean ± standard deviation (SD) or mean ± standard error (SE) as indicated in the figure legends, and were analyzed for significance using Student’s *t*-test. Data for grading of elastic fiber degradation were analyzed for significance using the nonparametric Mann-Whitney U-Test. Values of p < 0.05 were considered significant. Raw data on which statistical calculations were carried out are presented as Supporting Information.

## Results

### Brain arteries of *HtrA1*^*-/-*^ mice are normal

**Fig 1:**
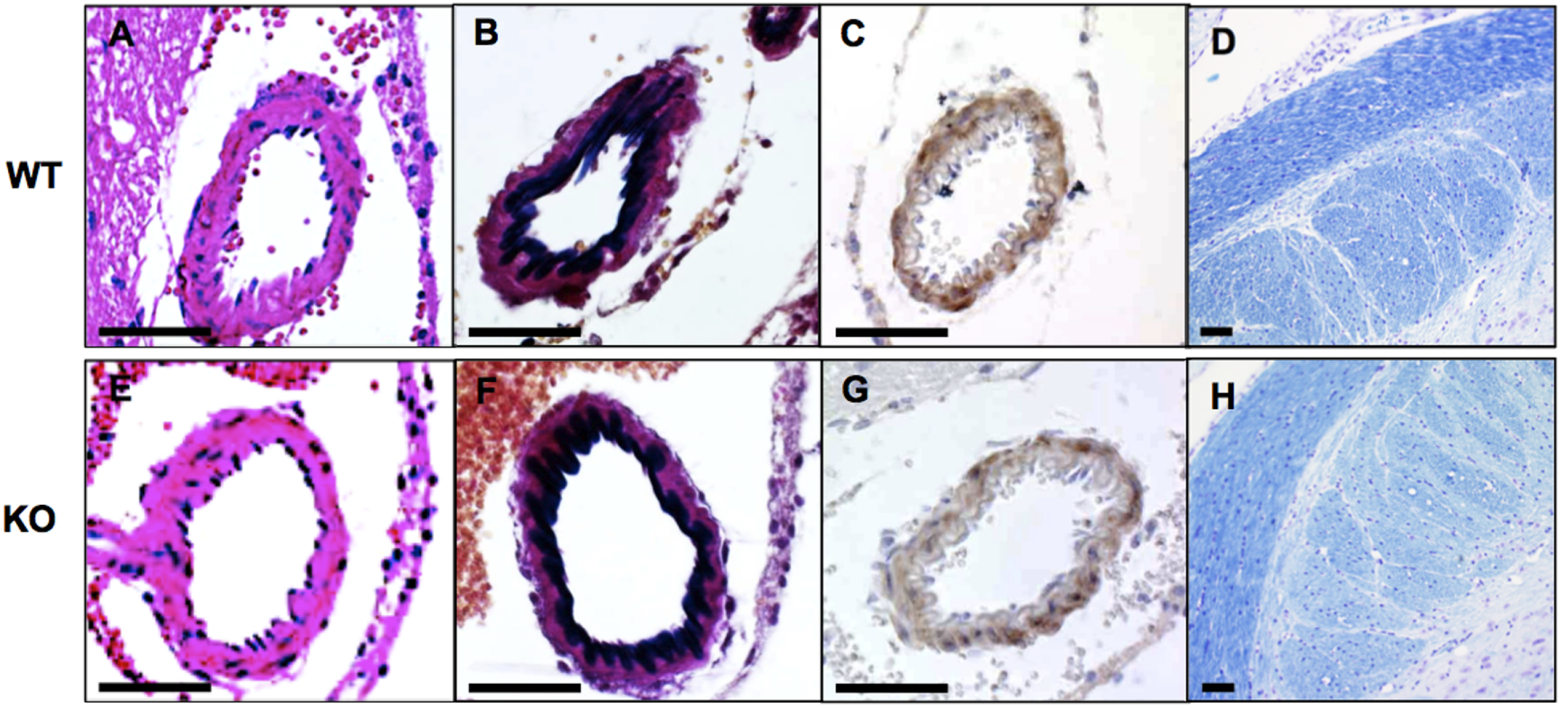
Histology of brain arteries of wild type and *HtrA1*^*-/-*^ mice. Coronal sections of midbrains from 52-week-old wild type (WT) and *HtrA1*^*-/-*^ (KO) mice from the 129/B6 background were stained with hematoxylin and eosin (A, E), elastica van Gieson for elastic fibers (B, F), anti-smooth muscle α-actin antibody, a VSMC marker (C, G), and luxol fast blue and cresyl violet for myelin (D, H). No obvious defects were observed in *HtrA1*^*-/-*^ brain arteries. Bars = 50 μm.

The Riken Mouse Clinic, a systematic and comprehensive phenotyping platform in Japan (http://ja.brc.riken.jp/la/jmc/mouse_clinic/m-strain/phenopub_top.html), detected no abnormality in *HtrA1*^*-/-*^ mice in various tests, including behavioral neurological assessments, except for a slight decrease in body mass index. *HtrA1*^*-/-*^ mice showed no clear defect in brain arteries, including the middle cerebral artery with diameter around 50–100 μm, even in old (52-week-old) mice (Fig 1). HE staining or immunostaining for smooth muscle α-actin (SMA) showed no apparent loss of VSMCs in *HtrA1*^*-/-*^ mouse brain arteries (Fig 1E and 1G). The intima of *HtrA1*^*-/-*^ mouse brain arteries appeared normal and the internal elastic lamina remained intact (Fig 1F). Klüver-Barrera staining, which stains myelin blue, did not reveal signs of leukoencephalopathy in the *HtrA1*^*-/-*^ mouse brain (Fig 1H).

### Altered structure of the *HtrA1*^*-/-*^ mouse aorta

Brain arteries of 100–1,000 μm in diameter are preferentially damaged in human CARASIL [28]. Mouse brain arteries are smaller than this size, whereas mouse aorta is 500–1,000 μm in diameter and has structures similar to human small brain arteries. Moreover, mouse HtrA1 is expressed not only in small brain arteries but also in the aortic media (Figure B-C in S1 Fig). We therefore assumed that the aorta of the *HtrA1*^*-/-*^ mouse would exhibit detectable abnormalities.

**Fig 2:**
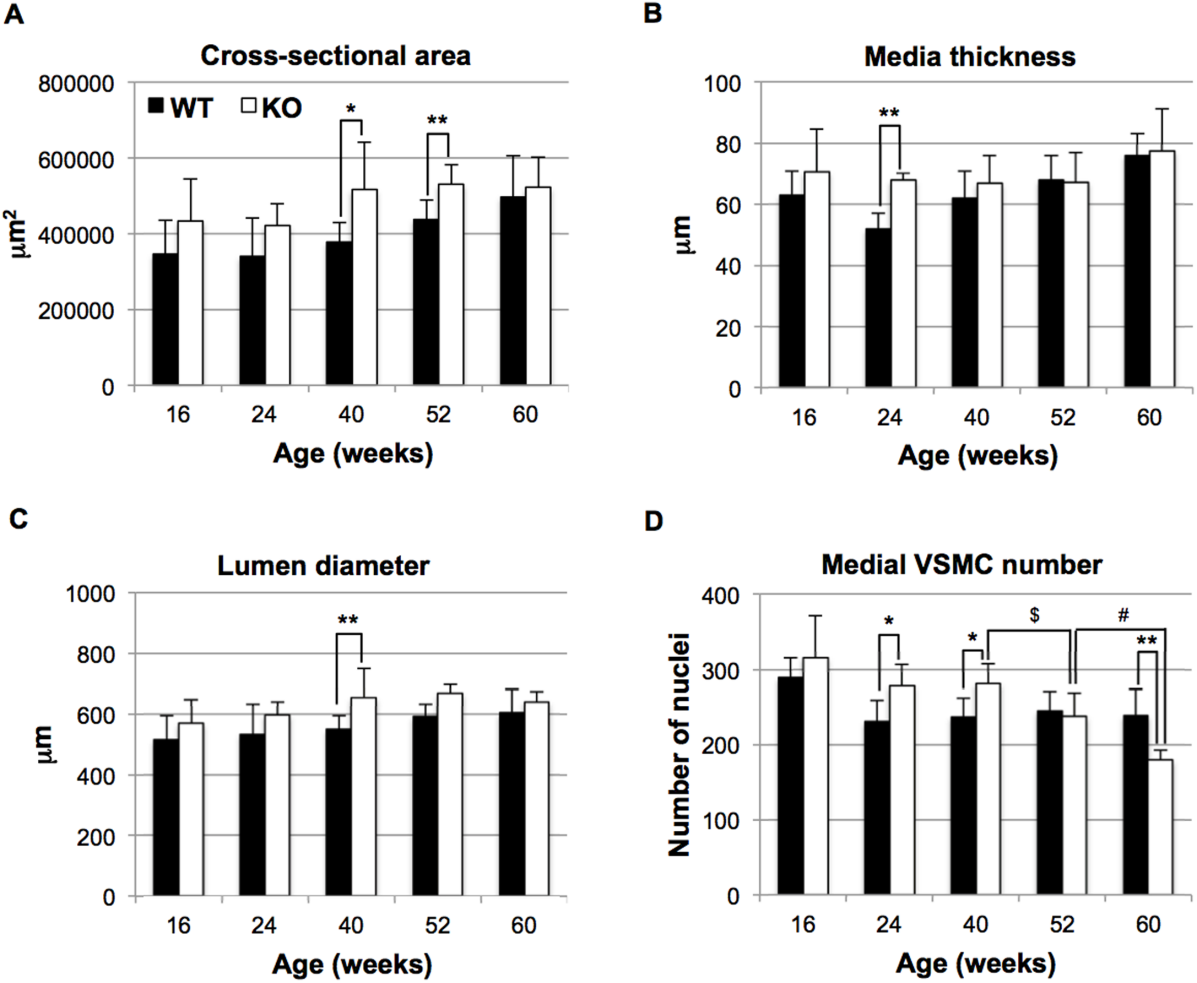
Histomorphometric parameters of aortas isolated from *HtrA1*^*-/-*^ mice. Cross sections from the upper half of the descending aorta were stained with elastica van Gieson (EVG), hematoxylin and eosin (HE), or picrosirius red. The EVG-stained sections were analyzed for **A**, cross-sectional area; **B**, media thickness; **C**, lumen diameter; the HE-stained sections were analyzed for **D**, medial VSMC number. Bars represent means ± SD (four to 10 mice were used for each group and 3–8 aorta sections per mouse were analyzed. The details are described in S2 File). Statistical significance was determined by Student’s *t*-test. Asterisks show significance between wild type (WT; black bars) and *HtrA1*^*-/-*^ (KO; white bars) mice at the same age (*, p <0.05; **, p <0.01). $ or # show significance (p <0.05) of the difference in medial VSMC numbers between 40- and 52-week-old, or 52- and 60-week-old, *HtrA1*^*-/-*^ mice, respectively.

Morphometric analysis of the aorta showed that the cross-sectional area, medial thickness, and lumen diameter were greater, though not always significantly, in *HtrA1*^*-/-*^ mice than in WT mice (Fig 2A–2C) at most time points during 16 to 60 weeks of age. In particular, *HtrA1*^*-/-*^ mice of 40 and 52 weeks old displayed significantly increased cross-sectional area, which represents aorta size and correlates inversely with contractility of the vessel (Fig 2A).

Loss of VSMCs is the earliest and most distinct event in CARASIL brain arteries [24, 27–29]. The number of VSMCs in *HtrA1*^*-/-*^ aortas, however, was increased at 16 weeks of age, and significantly higher than that in WT aortas at 24 and 40 weeks of age (Fig 2D). The aortic VSMC number usually decreases as the mouse ages [39]. The decrease in VSMC number was faster in *HtrA1*^*-/-*^ mice than in WT mice after 40 weeks. Consequently, the VSMC numbers in *HtrA1*^*-/-*^ mice became similar to those of WT mice at 52 weeks, and then became significantly lower than those of WT mice at 60 weeks. Staining with anti-PCNA showed that cell proliferation was increased, though not significantly, in *HtrA1*^*-/-*^ aortas at 40 weeks of age (Figure A in S2 Fig), whereas WT and *HtrA1*^*-/-*^ aortas showed comparable percentages of TUNEL-positive cells at 52 weeks of age (Figure B in S2 Fig).

### Loss of HtrA1 shifts isolated VSMCs to synthetic phenotype

The increase and then decrease in the number of VSMCs in the *HtrA1*^*-/-*^ mouse aorta during aging prompted us to analyze the properties of VSMCs. We established primary culture VSMCs from aortas of 10-week-old WT and *HtrA1*^*-/-*^ mice. VSMCs were isolated as cells that migrated out of minced pieces of the aortic media on the culture dishes. Cells that migrated from the medial pieces of *HtrA1*^*-/-*^ mouse aortas spread faster than cells from those of WT mouse aortas (Figure A in S3 Fig), suggesting that *HtrA1*^*-/-*^ VSMCs in vivo had shifted to the synthetic phenotype.

To confirm the synthetic modulation, we next evaluated marker protein expression in VSMCs using SMA and calponin as contractile markers and vimentin and osteopontin as synthetic markers. *HtrA1*^*-/-*^ VSMCs expressed less SMA but more vimentin and osteopontin than WT VSMCs (Figure C-D in S3 Fig). Although calponin is a contractile marker, it was expressed at high levels in *HtrA1*^*-/-*^ VSMCs. Since the expression patterns of three out of four markers agreed with characteristics of synthetic VSMCs, we postulated that *HtrA1*^*-/-*^ VSMCs were in the synthetic phenotype.

**Fig 3:**
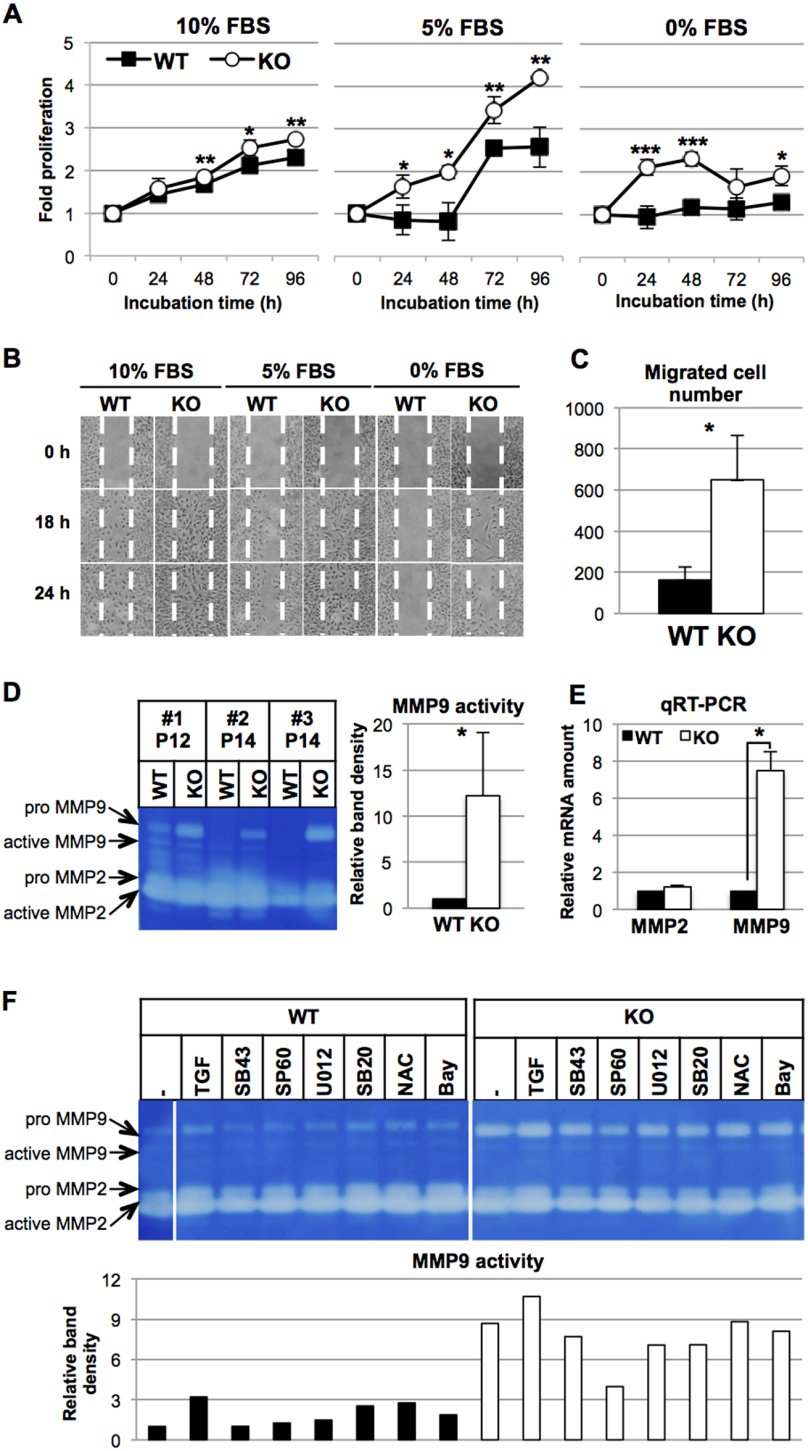
Phenotypic characterization of isolated *HtrA1*^*-/-*^ mouse VSMCs. **(A)** Rapid proliferation of *HtrA1*^*-/-*^ VSMCs. *HtrA1*^*-/-*^ (KO) and wild type (WT) VSMCs were cultured in medium containing 10%, 5%, or 0% FBS as indicated. Cell proliferation was assayed at the indicated time points (0 h was set at 15 h after plating). Points in the graphs represent means ± SD (n = 3–4). The experiments were carried out independently for three different batches of WT and *HtrA1*^*-/-*^ VSMCs at the matching passage. The data shown are a representative result from cells at passage 7. **(B-C)** Rapid migration of *HtrA1*^*-/-*^ VSMCs. **(B)** Cell migration was analyzed by the wound-healing assay. Cells were cultured in medium containing 10%, 5%, or 0% FBS as indicated. Photographs were taken at the indicated time points. White dotted lines indicate boundaries of the initial wounded area. The experiment was repeated three times and representative results are shown. Cells at passage 13 were used. **(C)** Cell migration was analyzed by a modified Boyden chamber assay. Cells were cultured in the chamber in medium containing 10% FBS for 24 h. The number of cells that migrated through the membrane was counted after DAPI staining. The bars are means ± SD (n = 3). The experiment was repeated twice times and representative results are shown. Cells at passage 12 were used. **(D-E)** Production of MMPs by isolated *HtrA1*^*-/-*^ mouse VSMCs. **(D)** Increased MMP9 activity in the culture media of *HtrA1*^*-/-*^ VSMCs. WT and *HtrA1*^*-/-*^ VSMCs were cultured in medium containing 0.5% FBS. After 24 h, the culture medium was recovered. The cells were also harvested, and cell lysates were analyzed by Western blot for tubulin content. The media, whose volumes were normalized by tubulin, were loaded onto a zymography gel. Three different batches of WT and *HtrA1*^*-/-*^ VSMCs at the matching passage (passage 12, P12 or 14, P14) were analyzed. The zymogram was analyzed by densitometer and the results are presented in the bar graph on the right. MMP9 activity of WT VSMCs was set to 1, and the relative MMP9 activity of *HtrA1*^*-/-*^ VSMCs is shown as mean ± SD. **(E)** MMP mRNA expression in WT and *HtrA1*^*-/-*^ VSMCs. RNA was extracted from cells that were harvested as described in D. MMP2 and MMP9 mRNA were measured by quantitative RT-PCR and normalized with GAPDH. The relative expression levels in *HtrA1*^*-/-*^ VSMCs were calculated using the levels of MMP2 and MMP9 mRNA in WT VSMCs as 1. Values represent mean ± SD (n = 3). Cells at passage 10 were used. Statistical significance in A, C, D, and E was determined by Student’s *t*-test. *; p <0.05. **; p < 0.01. ***; p <0.001. **(F)** Effects of TGF-β1, a radical scavenger, or signaling inhibitors on MMP9 activity of WT and *HtrA1*^*-/-*^ VSMCs. VSMCs were cultured in medium containing 0.1% FBS and treated for 24 h with various reagents as indicated. The culture supernatants were recovered and applied to zymography gels as described in D. The same samples (culture supernatants of untreated WT and *HtrA1*^*-/-*^ VSMCs) were applied to both gels and used as standards to compare activities on the separate gels. The uncropped zymograph pictures are shown in S4 Fig. MMP9 activity in the culture supernatant of untreated WT was set to 1 (leftmost lane) and relative activities for treated WT and *HtrA1*^*-/-*^ VSMCs were calculated and are presented in the bar graph in the lower panel. Cells at passage 14 were used. TGF = TGF-β1. SB43 = SB431542, a TGF-βR1 antagonist. SP60 = SP600125, a JNK inhibitor. U012 = U0126, a MEK1/2 (ERK1/2 upstream) inhibitor. SB20 = SB203580, a p38 MAPK inhibitor. NAC = N-acetylcysteine, a ROS scavenger. Bay = Bay11-7082, an NF-κB inhibitor. Black bars represent WT VSMCs; white bars represent *HtrA1*^*-/-*^ VSMCs.

Rapid proliferation and fast migration are major characteristics of synthetic VSMCs. *HtrA1*^*-/-*^ VSMCs proliferated faster than WT VSMCs in all media containing different concentrations (0, 5, and 10%) of FBS (Fig 3A). Furthermore, *HtrA1*^*-/-*^ VSMCs migrated faster than WT VSMCs in wound-healing assays under the different FBS concentrations (Fig 3B). The modified Boyden chamber assay in medium containing 10% FBS also showed that three times or more *HtrA1*^*-/-*^ VSMCs migrated to the bottom side of the chamber than WT VSMCs (Fig 3C). The expression patterns of the VSMC markers together with the rapid cell proliferation and robust migration indicated that *HtrA1*^*-/-*^ VSMCs are highly synthetic.

### High MMP9 activity in the culture media of *HtrA1*^*-/-*^ mouse VSMCs

MMPs play important roles in promoting cell migration [40]. MMP9 activities in the culture media of all three batches of the *HtrA1*^*-/-*^ VSMCs were more than 10 times higher than those of corresponding batches of WT VSMCs (Fig 3D). On the other hand, MMP2 activities were similar between *HtrA1*^*-/-*^ and WT VSMCs. Consistent with the gelatin zymography, we observed higher MMP9 mRNA levels in *HtrA1*^*-/-*^ VSMCs than in WT cells, but similar levels of MMP2 mRNA in these cells (Fig 3E). The increase in MMP9 activity of *HtrA1*^*-/-*^ VSMCs is hence caused mostly by an increase in transcription.

### Signal transduction pathway that induces MMP9 activity of *HtrA1*^*-/-*^ VSMCs

We next tried to identify the signal transduction pathway that induced the MMP9 activity of *HtrA1*^*-/-*^ VSMCs. VSMCs were treated with TGF-β1, various inhibitors of signal transduction, or a reactive oxygen species (ROS) scavenger (Fig 3F). TGF-β is known to induce MMP9 in VSMCs [41]. TGF-β1 induced secreted MMP9 activity 3-fold in WT VSMCs, but only 1.2-fold in *HtrA1*^*-/-*^ VSMCs. An inhibitor of TGF-βR1 (SB431542) did not decrease the MMP9 activity of *HtrA1*^*-/-*^ VSMCs. These data indicated that TGF-β signaling was not activated in *HtrA1*^*-/-*^ VSMCs, at least at the receptor level. NF-κB and AP-1 are the main transcription factors for MMP9 expression in VSMCs [42]. An inhibitor (Bay11-7082) of NF-κB did not affect the MMP9 activity of either WT or *HtrA1*^*-/-*^ VSMCs. Among the MAP kinase inhibitors examined, only an inhibitor (SP600125) of JNK, which is upstream of AP-1, suppressed the MMP9 activity of *HtrA1*^*-/-*^ VSMCs but not that of WT VSMCs, indicating that the JNK pathway was activated in *HtrA1*^*-/-*^ VSMCs (Fig 3F). JNK also serves as a signal transducer of oxidative stress [43]. A ROS scavenger, N-acetylcysteine (NAC), however, did not affect MMP9 activity in either WT or *HtrA1*^*-/-*^ VSMCs. Besides, inhibitors of p38 MAP kinase (SB203580) and MEK1/2 (U0126), two kinases that are downstream transducers of ROS signaling, did not suppress the MMP9 activity of *HtrA1*^*-/-*^ VSMCs. These data suggested that the MMP9 activity was elevated by stress other than oxidative stress. The increased migration of *HtrA1*^*-/-*^ VSMCs was specifically inhibited by the JNK inhibitor (SP600125), but not by the inhibitors of TFG-βR1, MEK1/2, p38, or NF-κB (S5 Fig). This result supports the view that the increased migration of *HtrA1*^*-/-*^ VSMCs is mainly due to the production of MMP9 induced through the JNK pathway.

### Effects of PDGF-BB or IGF-1 on proliferation, migration, and MMP9 activity

**Fig 4:**
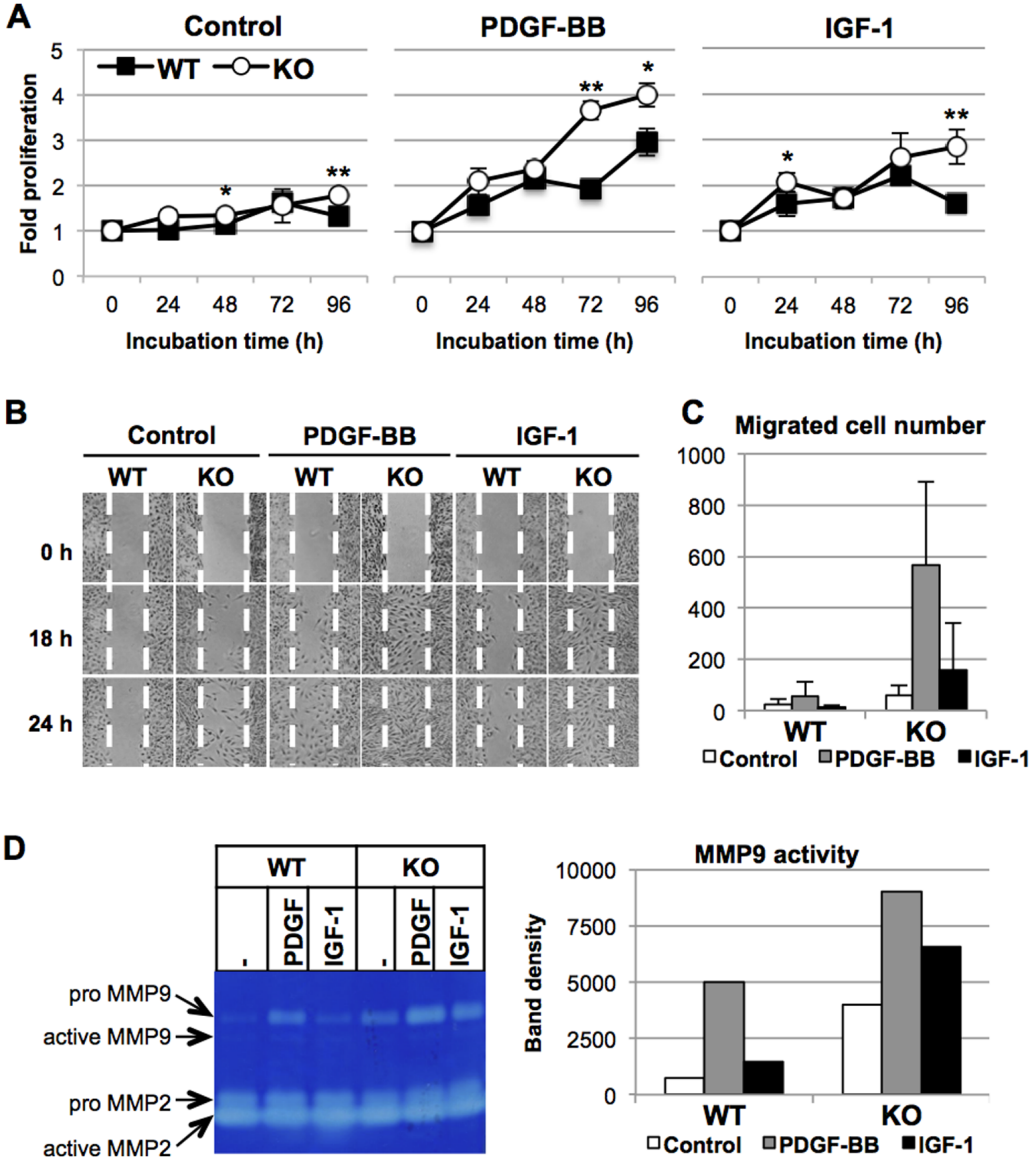
Effects of PDGF-BB or IGF-1 on proliferation, migration, and MMP activities of wild type and *HtrA1*^*-/-*^ mouse VSMCs. **(A)** Effects of PDGF-BB or IGF-1 on VSMC proliferation. Wild type (WT) and *HtrA1*^*-/-*^ (KO) VSMCs were cultured in medium containing 0.5% FBS with or without 20 ng/ml PDGF-BB or 10 ng/ml IGF-1, as indicated. Cell proliferation was assayed at the indicated time points (0 h was set at 15 h after plating). Points represent means ± SD (n = 3–4). The experiments were carried out independently for three different batches of WT and *HtrA1*^*-/-*^ VSMCs at the matching passage. The data shown are a representative result from cells at passage 7. Statistical significance was determined by Student’s *t*-test. *; p <0.05. **; p < 0.01. **(B-C)** Effects of PDGF-BB or IGF-1 on VSMC migration. **(B)** Cell migration was analyzed by the wound-healing assay. WT and *HtrA1*^*-/-*^ VSMCs were cultured in medium containing 0.5% FBS with or without 20 ng/ml PDGF-BB or 10 ng/ml IGF-1, as indicated. Photographs were taken at the indicated time points after wounding. Dotted white lines indicate the borders of the initial wounded area. The experiment was repeated three times and representative results are shown. Cells at passage 13 were used. **(C)** Cell migration was analyzed by the modified Boyden chamber assay. Cells were cultured in the assay chamber in medium containing 0.5% FBS with or without 20 ng/ml PDGF-BB or 10 ng/ml IGF-1, as indicated, for 24 h. The number of cells that migrated to the bottom side of the chamber was counted after DAPI staining. Data shown are means ± SD (n = 3). The experiment was repeated twice and representative results are shown. Cells at passage 9 were used. **(D)** Effects of PDGF-BB or IGF-1 on MMP9 activity of VSMCs. WT and *HtrA1*^*-/-*^ VSMCs were cultured for 72 h in medium containing 0.5% FBS without (- or Control) or with 20 ng/ml PDGF-BB (PDGF) or 10 ng/ml IGF-1, as indicated. The culture supernatants were recovered and applied to the zymography gel in volumes adjusted for the tubulin content in the cell lysates. Bands of MMP9 were analyzed by densitometer and are presented in the graph in the right panel. The experiment was repeated twice and representative results are shown. Cells at passage 12 were used.

Growth factors affect the phenotypic modulation of VSMCs. In general, PDGF-BB induces the synthetic phenotype and IGF-1 maintains the contractile phenotype [44]. We then examined effects of PDGF-BB or IGF-1 on the proliferation, migration, and MMP activities of VSMCs. PDGF-BB induced proliferation of both WT and, more strongly, *HtrA1*^*-/-*^ VSMCs (Fig 4A). IGF-1 also stimulated proliferation of both *HtrA1*^*-/-*^ and WT VSMCs, although its effect was weaker than that of PDGF-BB. PDGF-BB induced migration of WT VSMCs weakly (Fig 4B, left and middle panels), but it strongly induced migration of *HtrA1*^*-/-*^ VSMCs. Similarly, IGF-1 induced migration of WT VSMCs only slightly, but stimulated that of *HtrA1*^*-/-*^ VSMCs strongly (Fig 4B, right panels). Similar results were obtained with the modified Boyden chamber assay (Fig 4C). The number of *HtrA1*^*-/-*^ VSMCs that migrated through the membrane in the absence of growth factors was about three times higher than that of WT VSMCs. The PDGF-BB treatment increased the migration of *HtrA1*^*-/-*^ VSMCs 10-fold, but the migration of WT VSMCs only 2.5-fold. In contrast to the 2.5-fold stimulation of *HtrA1*^*-/-*^ VSMC migration by IGF-1, it did not induce migration of WT VSMCs.

We next examined the effects of PDGF-BB or IGF-1 on MMP activities (Fig 4D). In agreement with the migration assays, treatment with PDGF-BB highly induced MMP9 activity in the culture media of WT and *HtrA1*^*-/-*^ VSMCs, whereas IGF-1 only moderately induced MMP9 activity of both WT and *HtrA1*^*-/-*^ VSMCs. MMP2 activity was not affected by PDGF-BB or IGF-1.

### Loss of *HtrA1*^*-/-*^ induces VSMC death under oxidative stress

**Fig 5:**
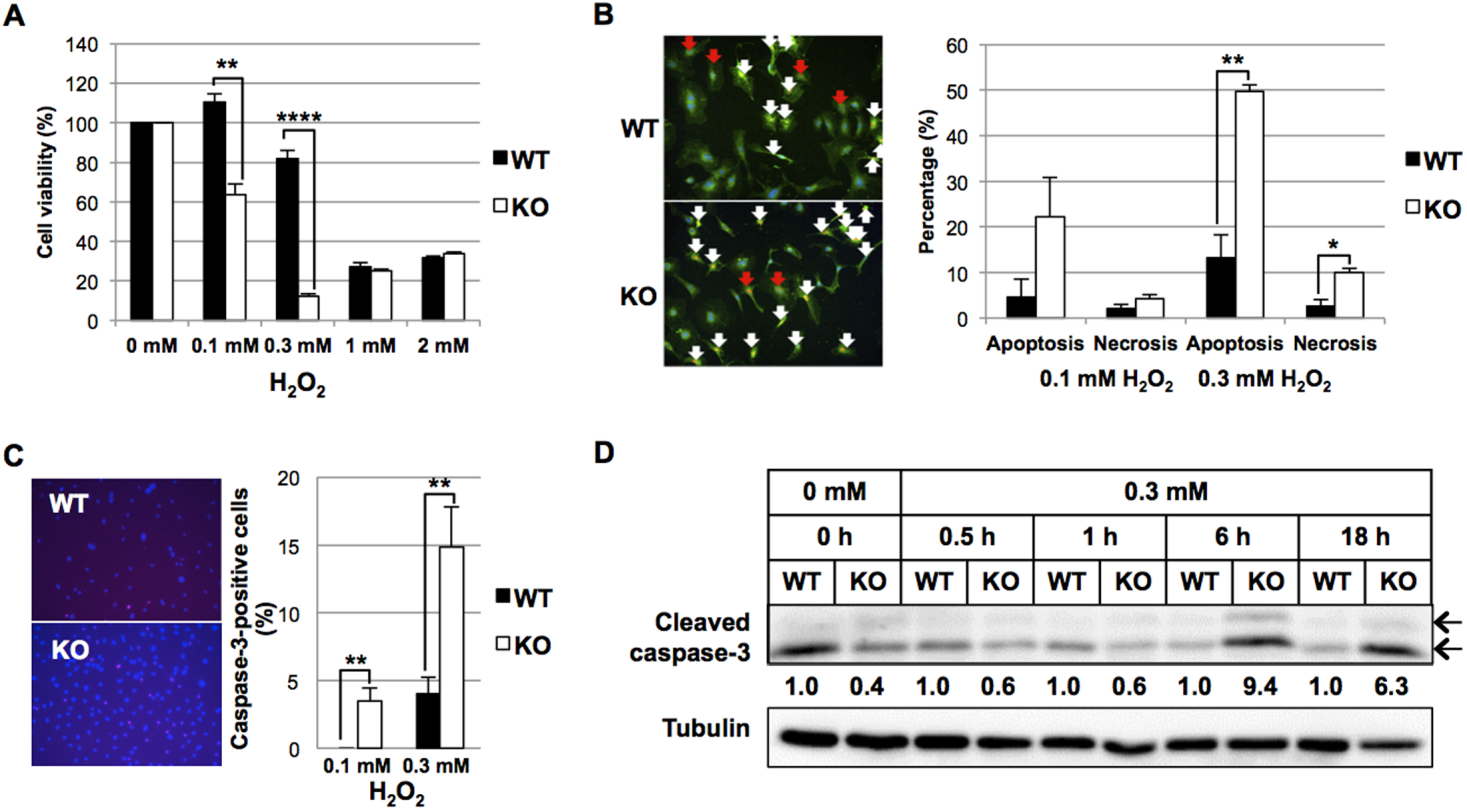
Effect of oxidative stress on wild type and *HtrA1*^*-/-*^ mouse VSMCs. Wild type (WT) and *HtrA1*^*-/-*^ (KO) VSMCs at passage 10 were starved in medium containing 0.1% FBS for 24 h. **(A)** Effect of H_2_O_2_ on cell viability of WT and *HtrA1*^*-/-*^ mouse VSMCs. Cells were treated with the indicated concentrations of H_2_O_2_ for 24 h in medium containing 0.1% FBS. Cell viability was examined using Cell Count Reagent SF (Nacalai Tesque). **(B)** Effect of H_2_O_2_ on cell death of WT and *HtrA1*^*-/-*^ mouse VSMCs. Cells were treated with 0.1 or 0.3 mM H_2_O_2_ for 6 h in medium containing 0.1% FBS. Cells were triply stained with Apoptotic/Necrotic/Healthy Cells Detection Kit (PromoKine) as follows: Hoechst 33342 (blue) indicating the entire cell population, FITC-Annexin V (green) indicating apoptotic cells and Ethidium homodimer III (red) indicating necrotic cells. Representative images of merged data are shown in the left panel. Apoptotic (white arrows) and necrotic (red arrows) cells were counted, and their percentages of the total cell number were calculated and are presented in the bar graph in the right panel. **(C)** Effect of H_2_O_2_ on apoptosis of WT and *HtrA1*^*-/-*^ mouse VSMCs. Cells were treated with 0.1 or 0.3 mM H_2_O_2_ for 6 h in medium containing 0.1% FBS. Cells were then immunostained with anti-cleaved caspase-3 antibody. Representative images of WT and *HtrA1*^*-/-*^ VSMCs treated with 0.3 mM H_2_O_2_ are shown in the left panel. Caspase-3-positive cells were counted, and their percentages of the total cell number were calculated and are presented in the bar graph in the right panel. **(D)** Effect of H_2_O_2_ on the expression of cleaved caspase-3 on WT and *HtrA1*^*-/-*^ mouse VSMCs. WT and *HtrA1*^*-/-*^ VSMCs were treated with 0.3 mM H_2_O_2_ in medium containing 0.1% FBS. Cell lysates were prepared at the time points indicated, separated by SDS- PAGE, and analyzed by Western blot for cleaved caspase-3. Band density was analyzed by densitometry. The expression levels were normalized with tubulin, and relative expression levels were calculated and are shown below the upper panel. The anti cleaved caspase-3 antibody detected the large fragments (17/19 kDa, indicated by arrows). Band densities of both were analyzed by densitometry, and summated. Bars in A, B, and C represent means ± SE (n = 3). Statistical significance was determined by Student’s *t*-test. *; p <0.05. **; p <0.01. ****; p < 0.0001.

Loss of VSMCs is the main characteristic of human CARASIL arteries [24, 28]. We have reported that HtrA1 deficiency made mouse embryonic fibroblast cells prone to oxidation-induced cell death [23], and we therefore examined the effect of H_2_O_2_ on cell death of VSMCs. As anticipated, low concentrations (0.1 and 0.3 mM) of H_2_O_2_ caused acute cytotoxicity in *HtrA1*^*-/-*^ VSMCs but not in WT VSMCs (Fig 5A). At 0.3 mM, H_2_O_2_ induced significantly higher incidence of apoptosis and necrosis in *HtrA1*^*-/-*^ VSMCs than in WT VSMCs, and the cells died mainly through apoptosis (Fig 5B). After treatment with 0.1 or 0.3 mM H_2_O_2_, more *HtrA1*^*-/-*^ VSMCs than WT VSMCs became cleaved caspase-3-positive (Fig 5C). Western blot analyses at various time points after H_2_O_2_ treatment showed that the expression of cleaved caspase-3 was higher in *HtrA1*^*-/-*^ VSMCs than in WT VSMCs after 6 h (Fig 5D). These data suggested that *HtrA1*^*-/-*^ VSMCs died more easily than WT VSMCs upon oxidative stress, and the cell death was mainly caused by apoptosis.

### Phenotypic shift of aortic VSMCs in vivo and histological abnormalities of *HtrA1*^*-/-*^ mouse aortas

**Fig 6:**
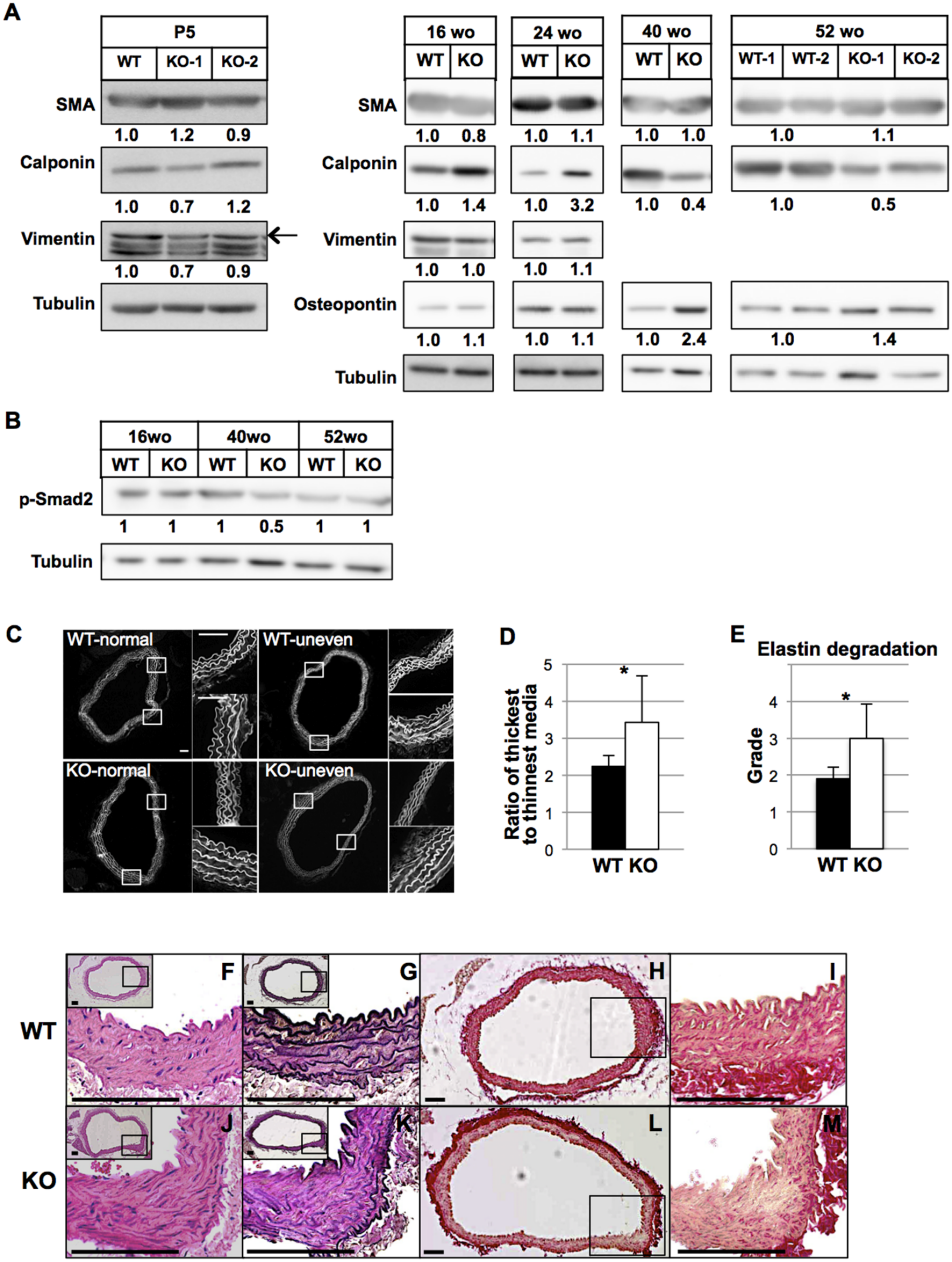
Characterization of in vivo VSMCs in the aorta of *HtrA1*^*-/-*^ mouse and histological abnormalities of aortas isolated from *HtrA1*^*-/-*^ mice. **(A)** Expression of VSMC contractile and synthetic markers in aortas of wild type (WT) and *HtrA1*^*-/-*^ (KO) mice. The entire descending thoracic aortas from three or four mice of 5 days old (P5) or 16, 24, 40, or 52 weeks old (wo) were combined and tissue extracts were prepared. The extracts were separated by SDS-PAGE and Western blot analysis was performed with VSMC markers, as indicated. Expression levels were normalized with tubulin, and relative expression levels were calculated and are shown below each panel. Two different batches of aortic extract from P5 *HtrA1*^*-/-*^ mice (KO-1 and KO-2), 52-wo wild type mice (WT-1 and WT-2), and *HtrA1*^*-/-*^ mice (KO-1 and KO-2) were analyzed. The arrow in the P5 blots shows the vimentin band; bands below the arrow are nonspecific proteins. **(B)** Phosphorylation of Smad2 in aortas of WT and *HtrA1*^*-/-*^ mice. Tissue extraction, SDS-PAGE, and Western blot were carried out as described in A using an antibody against p-Smad2. Relative expression levels after tubulin normalization are shown below the upper panel. **(C-D)** Uneven thickening of aortic media of 52-wo *HtrA1*^*-/-*^ mice. Cross sections from the upper half of the descending aorta were stained with elastica van Gieson (EVG), hematoxylin and eosin (HE), or picrosirius red. Cross sections were stained with HE, and autofluorescence images were obtained with a fluorescence microscope to visualize elastin structures. Upper and lower panels show representative images of normal aorta or aorta with uneven thickening in WT and *HtrA1*^*-/-*^ mice, respectively **(C)**. Two small images on the right side of each large image show magnified (4x) views of the boxed areas and represent the thickest (lower panels) and the thinnest (upper panels) areas. Bars = 100 μm. Three adjacent sections were analyzed for each aorta, and the incidence of uneven thickening and the ratio of thickest-to-thinnest media were calculated **(D)**. Bars in D represent means ± SD (WT, n = 10; *HtrA1*^*-/-*^, n = 8). Statistical significance was determined by Student’s *t*-test. *; p <0.05. **(E)** Elastic fiber degradation in the aortic media of 52-wo *HtrA1*^*-/-*^ mice. Grading of elastic fiber degradation was carried out using autofluorescence images of HE-stained sections based on a reported method [45]; the details are described in the Supplementary methods in S1 File. Pictures depicting typical images of the WT and *HtrA1*^*-/-*^ aortas are shown in S6 Fig. Bars represent means ± SD (WT, n = 10; *HtrA1*^*-/-*^, n = 8). Statistical significance in E was determined by nonparametric U-test (Mann-Whitney). *; p <0.05. **(F-M)** Elastic fiber degradation and decrease in collagens in 52-wo *HtrA1*^*-/-*^ mouse aortas. Cross sections were stained with HE (F, J), EVG (G, K), or picrosirius red (H, L) for collagens. Insets in F, G, J, and K depict the entire cross sections, with boxed areas that are magnified in the main images. I and M show higher magnifications of the boxed areas of H and L, respectively. Bars = 100 μm.

We next examined the expression of synthetic and contractile marker proteins to evaluate the phenotypic characteristics of VSMCs in vivo. Aortas from 5-day-old *HtrA1*^*-/-*^ mice expressed SMA, calponin, and vimentin at similar levels to those from WT mice, indicating that the differentiation and maturation of VSMCs were not affected in *HtrA1*^*-/-*^ mice (Fig 6A, leftmost panels). *HtrA1*^*-/-*^ mouse aortas expressed more calponin than WT mouse aortas at 16 and 24 weeks of age, but both expressed comparable levels of SMA, osteopontin, and vimentin. Calponin levels in *HtrA1*^*-/-*^ mouse aortas decreased to about half of those in WT mouse aortas at 40 and 52 weeks of age, and aortas of *HtrA1*^*-/-*^ mice expressed osteopontin more strongly than WT mouse aortas (Fig 6A). The expression profile of these marker proteins at 40 and 52 weeks agrees with the notion that aortic VSMCs are in the synthetic phenotype.

The onset of CARASIL has been associated with modified TGF-β signaling [24]. The *HtrA1*^*-/-*^ mouse aortas, however, did not show either an increase or a decrease in the level of phosphorylated Smad2 (Fig 6B). Together with the data for isolated VSMCs (Fig 3F and S5 Fig), this result suggests that the TGF-β signaling pathway does not contribute to the early etiology of CARASIL.

The increase in MMP9 activity of *HtrA1*^*-/-*^ VSMCs may disrupt the elastic fibers and other ECM components in the aorta. The aortas of *HtrA1*^*-/-*^ mice exhibited uneven thickening of the media, and splitting or degradation of elastic fibers (Fig 6C–6E). Uneven medial thickening, which we defined as the thickest part of the aorta being 2.5 times or more as thick as the thinnest part, was observed in 17 of 24 aorta sections from eight 52-week-old *HtrA1*^*-/-*^ mice (71%), but only in five out of 30 aorta sections from ten age-matched WT mice (17%). The ratio of the thickest to the thinnest aortic media was significantly higher in 52-week-old *HtrA1*^*-/-*^ mice than in WT mice (Fig 6D). The elastic fibers of the *HtrA1*^*-/-*^ mouse aorta were fragmented and the interval between elastic fibers was increased. We used a modification of a reported grading system [45] to estimate elastin degradation semi-quantitatively (S6 Fig). The *HtrA1*^*-/-*^ mouse aortas at 52 weeks of age showed significantly more severe degradation than the WT mouse aortas at the same age (Fig 6E).

Fibrillar collagens are specifically decreased in the media of CARASIL arteries [28]. Collagens were also decreased in the aged *HtrA1*^*-/-*^ mouse aorta. Picrosirius red staining showed that collagen content was low in the entire area of the aortic media of *HtrA1*^*-/-*^ mice at 52 weeks of age. The adventitia of both *HtrA1*^*-/-*^ and WT aortas, however, stained equally strongly (Fig 6H and 6L). The lowest collagen staining coincided with the thickened areas of *HtrA1*^*-/-*^ aortas (Fig 6L, boxed area, and M).

All these data show that the aorta of the *HtrA1*^*-/-*^ mouse recapitulates major abnormalities found in human CARASIL arteries: loss of VSMCs, elastic fiber disruption, and collagen depletion.

## Discussion

This study has revealed that the thoracic aorta of aged homozygous *HtrA1*^*-/-*^ mice shows pathological changes characteristic of those in the brain arteries of CARASIL patients [28]; among those changes is the loss of VSMCs (Fig 2D). The mouse aorta has a structure and diameter similar to the human small brain arteries that are preferentially affected in CARASIL. CARASIL patients frequently show mild sclerotic changes in the coronary arteries, and arteries of other visceral organs and subcutaneous tissues [26–29]. These findings suggest that CARASIL is a systemic vascular disorder, and the size, and hence the structure, of the artery may contribute to the pathological changes. The number of aortic VSMCs increased in young *HtrA1*^*-/-*^ mice (Fig 2D), but decreased rapidly after 40 weeks and became significantly lower than that in WT aortas at 60 weeks. The initial increase of VSMCs may be caused by synthetic modulation.

CARASIL patients show severe vascular abnormalities in their twenties. In contrast, the in vivo phenotype is weak even in aged *HtrA1*^*-/-*^ mice. One possible explanation for this discrepancy is a difference in the functional contribution of secretory HtrA family members in mouse and human vessels. HtrA1 may play the main role in human brain vessels, but not in mouse vessels, and HtrA3 or HtrA4 may compensate for the loss of HtrA1 in mouse vessels. Heterozygous mutations of *HTRA1* cause late onset SVD in humans [30]. The in vivo phenotype of *HtrA1*^*-/-*^ mice might thus mimic the abnormalities caused by heterozygous *HTRA1* mutations. Another possibility is the difference in ageing processes in human and mouse vessels. Lipid metabolism is different [46–48] and the telomeres are longer in mouse cells than human cells [47, 49]. Analysis of double knockout mice for *HtrA1* and *ApoE*, *Lrp1*, or *Terc* may give the answer to this possibility.

Primary cultures of aortic VSMCs established from *HtrA1*^*-/-*^ mice are highly synthetic and proliferate more quickly, migrate faster, and produce higher MMP9 activity than WT mouse VSMCs (S3 Fig and Fig 3A–3E). Responses of isolated VSMCs to PDGF-BB and IGF-1 support our conclusion that *HtrA1*^*-/-*^ VSMCs are skewed towards the synthetic phenotype (Fig 4A). The fast migration of *HtrA1*^*-/-*^ VSMCs may be ascribed to the high activity of MMP9, because both are inhibited by a JNK inhibitor (Fig 3F and S5 Fig). The high activity of MMP9 may also explain the elastic fiber degradation in the aorta (Fig 6E and S6 Fig). MMP9 plays critical roles in pathological processes of cardiovascular diseases that involve tissue remodeling, inflammation, or fibrosis [42]. The increased MMP9 activity of *HtrA1*^*-/-*^ mouse VSMCs should hence contribute heavily to the onset of aortic abnormalities. Examination of the MMP9 activity in human CARASIL arteries should give a good insight into the etiology of this disease.

This study highlights the synthetic modulation of VSMCs as the earliest key event caused by HtrA1 deficiency. The mechanisms that underlie the synthetic modulation of *HtrA1*^*-/-*^ VSMCs are unclear. One possible mechanism could be damage to elastic fibers, collagens, or other ECM molecules. CARASIL is a systemic disorder of the ECM; vertebral disc herniation and spondylosis deformans are major diagnostic signs of CARASIL, and patients frequently show limb arthropathy and keratotic skin changes. Changes in the vascular ECM are a major factor that induces synthetic modulation of VSMCs [33, 50]. Bunton *et al* [51] analyzed Marfan syndrome (MFS) model mice carrying a genetically disrupted *fibrillin 1* gene, and proposed that loss of connection between VSMCs and elastic fibers initiated the synthetic modulation of VSMCs. This modulation contributes to elastolysis due to overproduction of MMP9, and leads to eventual collapse of the vessel structure in this disease [51].

It is not clear what triggers the loss of VSMCs in the *HtrA1*^*-/-*^ mouse aorta at 52 weeks of age and older. One possibility is the enhanced migration of synthetic *HtrA1*^*-/-*^ VSMCs, which dislocates the VSMCs. More likely, however, the long-lasting synthetic state and stress accumulated therein might induce cell death. In support of this, we showed that primary cultures of *HtrA1*^*-/-*^ VSMCs are prone to cell death caused by oxidative stress (Fig 5). *HtrA1*^*-/-*^ VSMCs died mostly through apoptosis (Fig 5B–5D). Other types of stress, such as oxidized LDL treatment and endoplasmic reticulum (ER) stress induced by tunicamyicin or thapsigargin treatment, also promoted cell death of *HtrA1*^*-/-*^ VSMCs more strongly than that of WT VSMCs (C. Oka *et al*, unpublished data).

Apoptosis of VSMCs contributes substantially to the pathogenesis of cardiovascular diseases such as atherosclerosis, MFS, and cerebral autosomal dominant arteriopathy with subcortical infarcts and leukoencephalopathy (CADASIL). These diseases are associated with medial cystic degeneration, which is characterized by medial atrophy, VSMC loss, elastin fragmentation, increased glycosaminoglycans, and speckled calcification. Lines of evidence suggest that VSMC apoptosis is a primary and early event in these diseases, and it can alone trigger all of these secondary damages [52]. In atherosclerosis, VSMCs become highly mobile due to synthetic modulation and translocate to the intima, where apoptosis of VSMCs is activated by macrophages through death ligand-death receptor interaction [53]. Reduced extracellular deposition and altered quality of ECM proteins may play a role in VSMC apoptosis in MFS. Up-regulated MMP2 and caspases exteriorized from apoptotic VSMCs may contribute to the degradation of ECM proteins and subsequent VSMC apoptosis in MFS [54, 55]. Although TGF-β is known to heavily contribute to the pathogenesis of MFS, blockade of TGF-β signaling by losartan is not effective in preventing apoptosis of MFS VSMCs [56]. Rather, the p38 MAP kinase pathway appears to regulate apoptosis of MFS VSMCs. Loss of VSMCs through apoptosis is also prominent in the small arteries of the brain of CADASIL patients [57]. Mutated Notch3 extracellular domain peptides are aggregated and deposited on the surface of VSMCs. This abnormal deposition is toxic to cells and induces oxidative stress, ER stress, or mitochondrial dysfunction and subsequently triggers apoptosis of VSMCs [58, 59]. Apoptosis caused by altered interaction with the ECM, by activated MAP kinase pathways, or by various stress conditions may account for the decrease in aortic VSMCs in aged *HtrA1*^*-/-*^ mice. Zhang *et al* reported that activation of the JNK pathway promoted VSMC apoptosis [41]. We showed activation of the JNK pathway in *HtrA1*^*-/-*^ VSMCs (Fig 3F and S5 Fig). We could not detect a significant increase in TUNEL-positive VSMCs in 52-week-old *HtrA1*^*-/-*^ mouse aortas (Figure B in S2 Fig). Detection of apoptosis in vivo is usually difficult, however, particularly when the cell loss is a slow process [52].

We did not detect significant changes in the level of p-Smad2 in the aorta of *HtrA1*^*-/-*^ mice (Fig 6B). Increased TGF-β signaling has been reported as an etiologic factor of CARASIL [24]. In contrast, Beaufort *et al* [12] reported down-regulation of TGF-β signaling in CARASIL. Our current data suggest that the increase in the TGF-β signaling is not a primary event, but probably a late-stage event induced by extensive degradation of the ECM, which absorbs TGF-β, or by secondary inflammatory reactions.

Our previous study revealed that the PDZ domain of mouse HtrA1 binds to denatured C-terminal ends of C-propeptides of fibrous collagens, and this binding stimulates the protease activity of HtrA1 [60]. We proposed that mammalian HtrA1 functions in a protein quality control system for collagens and other ECM proteins in the secretion processes inside or in the vicinity of cells. Deficiency of HtrA1 may disturb the secretion of collagens and other ECM proteins, causing stressful conditions to accumulate inside the cell and leading to modulation of VSMC phenotypes. Impairment of the secretion of ECM proteins may also compromise cell-ECM interaction of VSMCs, further enhancing the phenotypic changes. Synthetic VSMCs produce high MMP9 activity that degrades the ECM, resulting in a vicious cycle that may lead to VSMC death and eventually to the onset of CARASIL.

Interestingly, HtrA1 levels are increased in the aorta of mice in which the low-density lipoprotein receptor-related protein 1 (*lrp1*) gene is deleted in smooth muscle cells (*smLRP1*^*-/-*^ mice) [61]. These mice have extensive aortic dilatation accompanied by elastic fiber degradation and medial thickening. Aortic VSMCs of *smLRP1*^*-/-*^ mice show a highly synthetic phenotype. Heterozygous mutations of *HTRA1* were recently reported in a late-onset familial SVD group [30]. These reports and our current work suggest that optimal proteolytic activity of HtrA1 is essential to maintain VSMCs in the contractile phenotype. Since the *HtrA1*^*-/-*^ mouse recapitulates the early events of the major vascular pathology of human CARASIL, it may be a valuable tool to reveal the molecular mechanisms underlying the vascular abnormality of this disease.

## Supporting information

S1 Fig HtrA1 and HtrA3 expression in small arteries and aortas. (PDF)

S2 Fig PCNA immunostaining and TUNEL staining of the *HtrA1*^*-/-*^ mouse aorta. (PDF)

S3 Fig Aortic vascular smooth muscle cell isolation. (PDF)

S4 Fig Effects of TGF-β1, a radical scavenger, or signaling inhibitors on MMP9 activity of wild type (WT) and *HtrA1*^*-/-*^ (KO) VSMCs. (PDF)

S5 Fig Effects of inhibitors of signal transduction pathways on *HtrA1*^*-/-*^ (KO) VSMC migration. (PDF)

S6 Fig Elastic fiber degradation in the aortic media of 52-wo *HtrA1*^*-/-*^ mice. (PDF)

S7 Fig Original uncropped blot of Fig 5D. (PDF)

S8 Fig Original uncropped blots of Fig 6. (PDF)

S1 File Supplementary methods. (PDF)

S2 File Supplementary data of Fig 2. (XLSX)

S3 File Supplementary data of Fig 3. (XLSX)

S4 File Supplementary data of Fig 4. (XLSX)

S5 File Supplementary data of Fig 5. (XLSX)

S6 File Supplementary data of Fig 6. (XLSX)

S1 Table Growth factors and inhibitors used for cell culture. (PDF)

S2 Table Forward (F) and reverse (R) primers used for qRT-PCR. (PDF)

S3 Table Primary antibodies used for immunostaining. (PDF)

S4 Table Primary antibodies used for Western blotting. (PDF)
