# Supplementary material for: Loss of HtrA1 serine protease induces synthetic modulation of aortic vascular smooth muscle cells
Source: PLoS One. 2018 May 16;13(5):e0196628. doi: 10.1371/journal.pone.0196628 (PMC5955505; doi:10.1371/journal.pone.0196628)
Supplement: S1 Fig — (PDF) [file pone.0196628.s001.pdf]

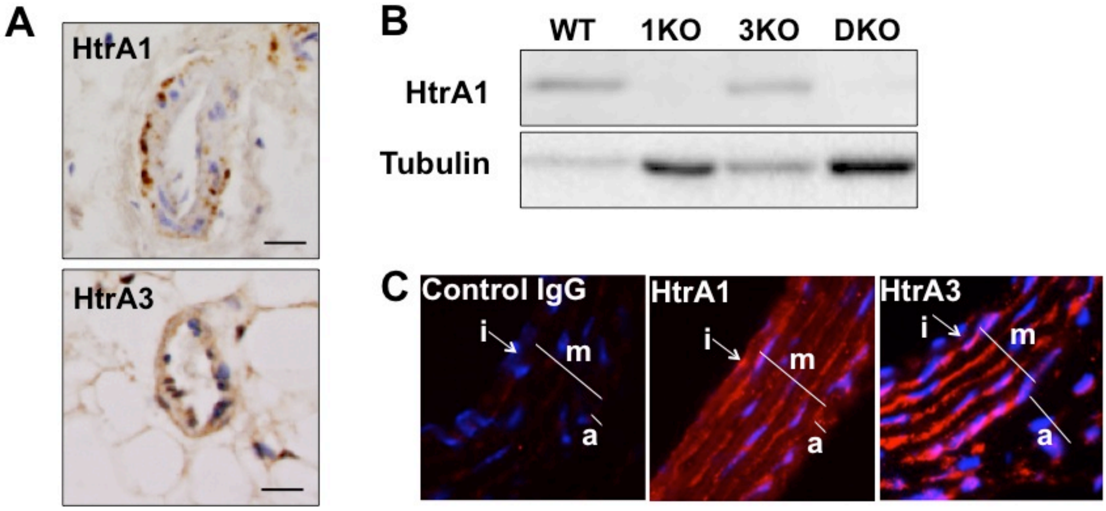

S1 Fig. HtrA1 and HtrA3 expression in small arteries and aortas. **(A)** Popliteal arteries from an adult mouse were stained with anti-HtrA1 or anti-HtrA3 antibody. Bars = 10 μm. **(B)** Western blot analysis of HtrA1 in the aorta of wild type (WT), *HtrA1*$^{-/-}$ (1KO), *HtrA3*$^{-/-}$ (3KO), and *HtrA1*$^{-/-}$;*HtrA3*$^{-/-}$ (DKO) mice. 129/B6 background mice at 52 weeks of age were used. **(C)** HtrA1 or HtrA3 staining of aorta from 8-week-old ICR mice. Aortas were immunostained with anti-HtrA1 (middle) or anti-HtrA3 (right) antibody and counterstained with DAPI (blue). HtrA1 and HtrA3 were expressed in VSMCs in the media. Staining without primary antibody was used as control (left). i=intima; m=media; a=adventitia.
