# Supplementary material for: Loss of HtrA1 serine protease induces synthetic modulation of aortic vascular smooth muscle cells
Source: PLoS One. 2018 May 16;13(5):e0196628. doi: 10.1371/journal.pone.0196628 (PMC5955505; doi:10.1371/journal.pone.0196628)
Supplement: S2 Fig — (PDF) [file pone.0196628.s002.pdf]

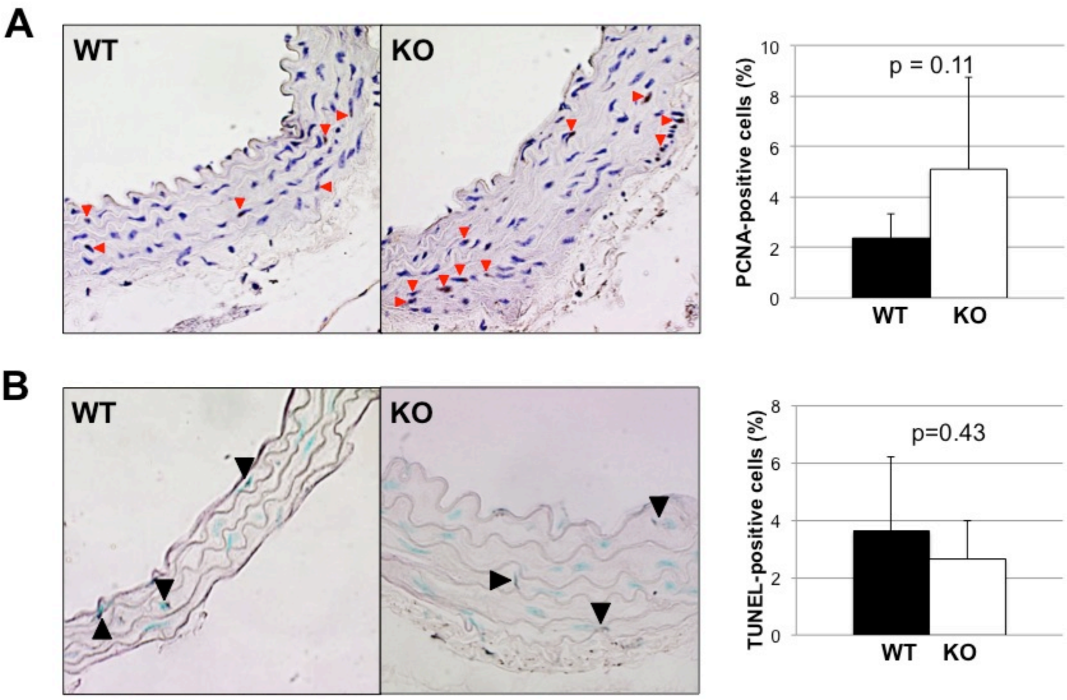

S2 Fig. PCNA immunostaining and TUNEL staining of the *HtrA1*$^{-/-}$ mouse aorta. **(A)** Cross-sectioned aorta tissues from 40-week-old wild type (WT) and *HtrA1*$^{-/-}$ (KO) mice were stained with anti-PCNA antibody and counterstained with hematoxylin. Red arrowheads indicate PCNA-positive cells, and percentages of PCNA-positive cells are shown in the graph on the right. Bars represent means ± SD (WT, n=6; KO, n=5). **(B)** Cross-sectioned aorta tissues from 52-week-old mice were stained with TUNEL and counterstained with methyl green. Arrowheads indicate TUNEL-positive cells, and percentages of TUNEL-positive cells are shown in the graph on the right. Bars represent means ± SD (n=6). p values were calculated by Student's *t*-test.
