# Supplementary material for: Loss of HtrA1 serine protease induces synthetic modulation of aortic vascular smooth muscle cells
Source: PLoS One. 2018 May 16;13(5):e0196628. doi: 10.1371/journal.pone.0196628 (PMC5955505; doi:10.1371/journal.pone.0196628)
Supplement: S3 Fig — (PDF) [file pone.0196628.s003.pdf]

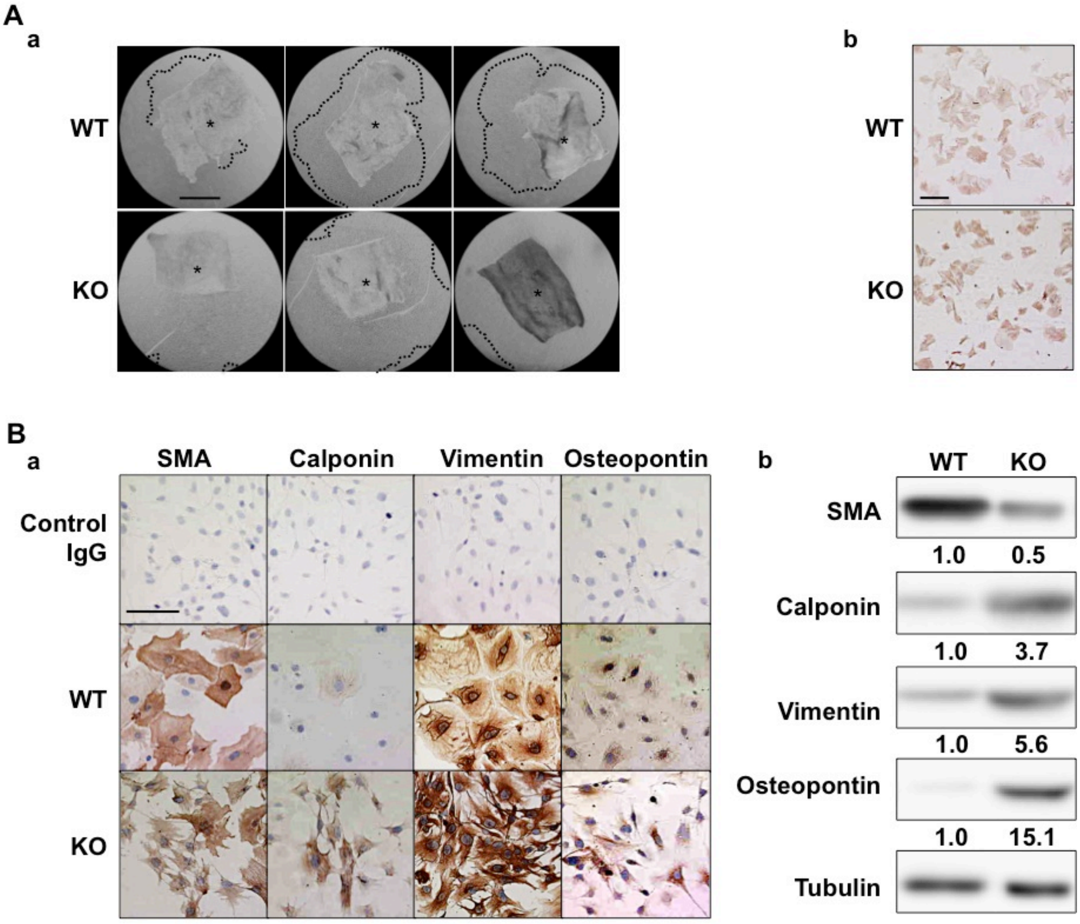

S3 Fig. Aortic vascular smooth muscle cell isolation. **(A)** Medial explants of wild type and *HtrA1*$^{-/-}$ aortas and SMA staining in wild type and *HtrA1*$^{-/-}$ VSMCs. **(a)** The aortic media isolated from 10-week-old wild type (WT) and *HtrA1*$^{-/-}$ (KO) mice were cut into pieces and placed in culture dishes. Cells that had migrated from the medial explants at day 14 are depicted. Dotted lines mark the forefront of migrated cells. Asterisks mark the medial explants. Scale bar=1 cm. **(b)** Expression of a smooth muscle-specific marker, SMA, in isolated WT and *HtrA1*$^{-/-}$ VSMCs at passage 6. Cells (1x10^4/well) were plated in a 6-well plate, cultured for 72 h, and immunostained for SMA (brown). Scale bar=100 µm. **(B)** Expression of contractile and synthetic markers in WT and *HtrA1*$^{-/-}$ VSMCs. **(a)** Immunostaining of VSMCs. Cells at passage 13 were immunostained for the indicated VSMC markers (brown) and counterstained with hematoxylin to visualize nuclei (blue). Staining without primary antibody served as control (top panels). Scale bar=100 µm. **(b)** Western blot analysis of VSMC markers. Cell lysates from passage 8. VSMCs were separated by SDS-PAGE and Western blot analysis was performed for the indicated VSMC markers.

The expression levels were normalized with tubulin, and relative expression levels were calculated and are shown below the panels.
