# Supplementary material for: Loss of HtrA1 serine protease induces synthetic modulation of aortic vascular smooth muscle cells
Source: PLoS One. 2018 May 16;13(5):e0196628. doi: 10.1371/journal.pone.0196628 (PMC5955505; doi:10.1371/journal.pone.0196628)
Supplement: S4 Fig — (PDF) [file pone.0196628.s004.pdf]

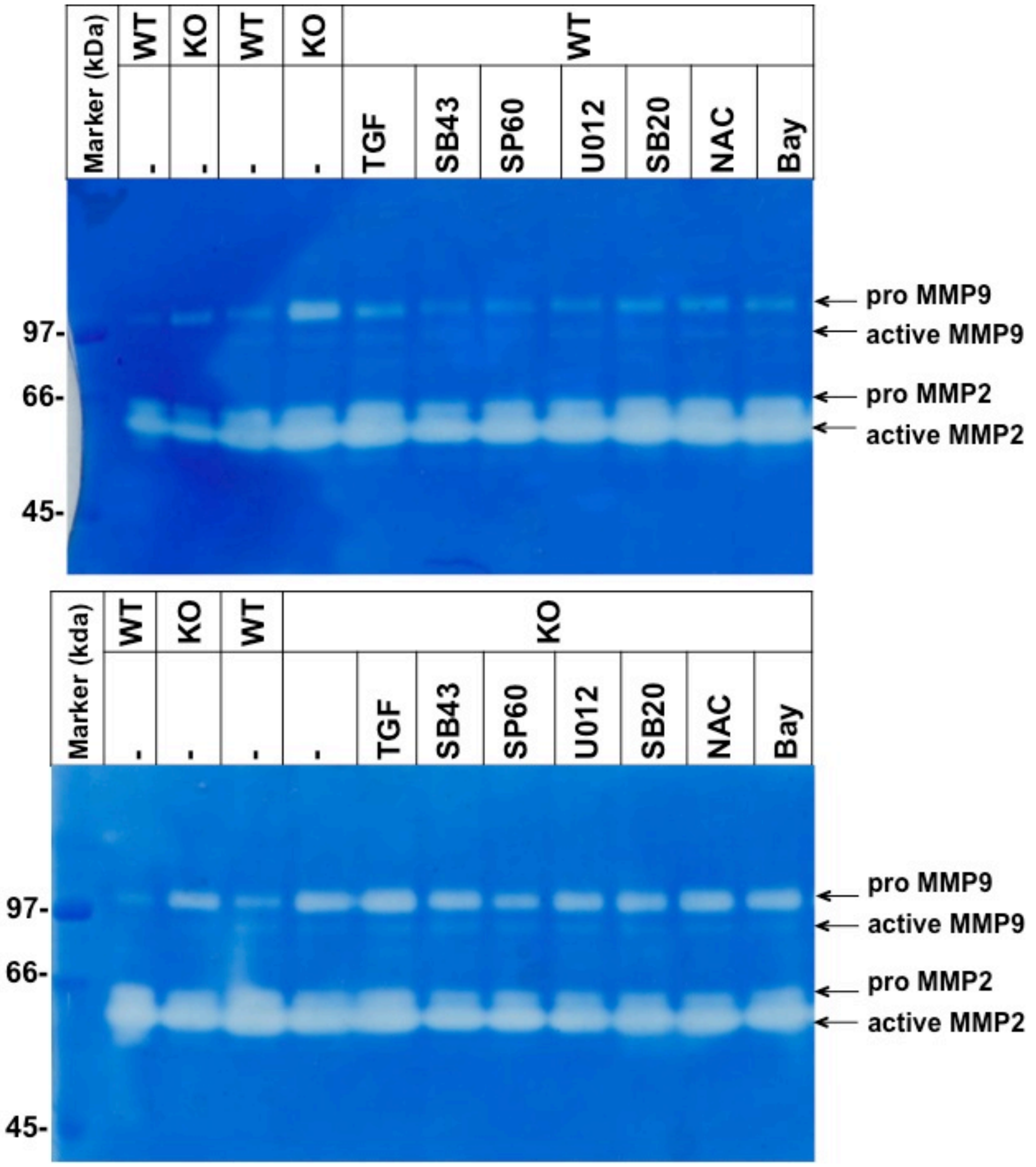

S4 Fig. Effects of TGF-β1, a radical scavenger, or signaling inhibitors on MMP9 activity of wild type (WT) and *HtrA1*$^{-/-}$ (KO) VSMCs. VSMCs were cultured in medium containing 0.1% FBS and treated for 24 h with various reagents as indicated. The culture supernatant was recovered and applied to a zymography gel as described in Fig 2D. The zymography was carried out in two separate gels due to the limitation of wells. The same samples of untreated (-) WT and untreated (-) *HtrA1*$^{-/-}$ were included in both gels (leftmost two lanes) as standards to compensate for any variation in proteolytic activity in the separate gels. TGF=TGFβ1. SB43=SB431542, a TGF-βR1 antagonist. SP60=SP600125, a JNK inhibitor. U012=U0126, a MEK1/2 (ERK1/2 upstream) inhibitor. SB20=SB203580, a p38 MAPK inhibitor. NAC=N-acetylcysteine, a ROS scavenger. Bay=Bay11-7082, a selective NF-κB inhibitor.
