# Supplementary material for: Loss of HtrA1 serine protease induces synthetic modulation of aortic vascular smooth muscle cells
Source: PLoS One. 2018 May 16;13(5):e0196628. doi: 10.1371/journal.pone.0196628 (PMC5955505; doi:10.1371/journal.pone.0196628)
Supplement: S5 Fig — (PDF) [file pone.0196628.s005.pdf]

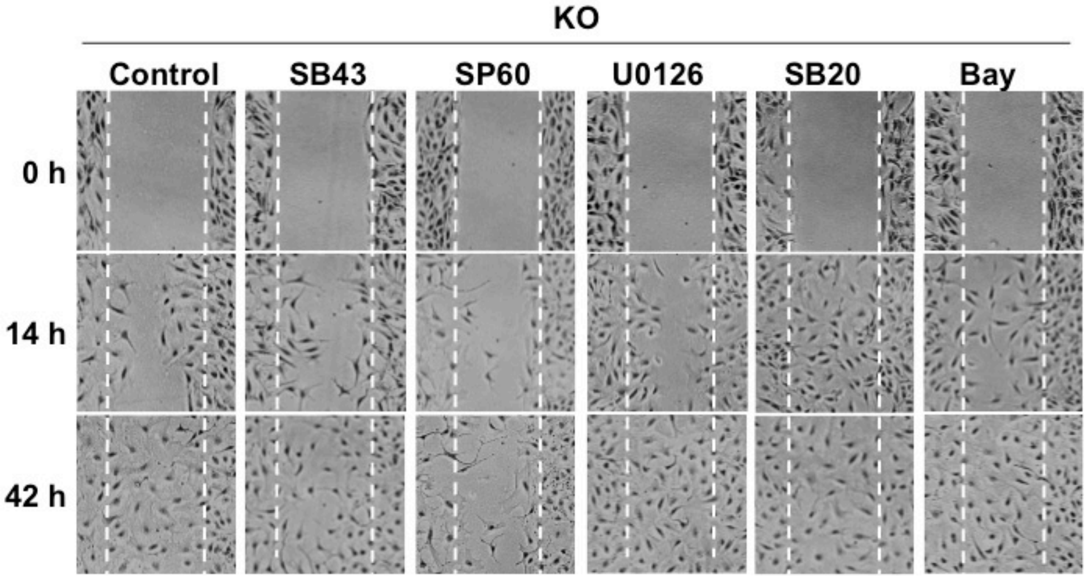

S5 Fig. Effects of inhibitors of signal transduction pathways on *HtrA1*$^{-/-}$ (KO) VSMC migration. Cell migration was analyzed by the wound-healing assay. Cells were cultured in medium containing 0.5% FBS without (Control) or with inhibitors as indicated. Photographs were taken at the indicated time points. Dotted white lines indicate the borders of the initial wounded area. The experiment was repeated three times and representative results are shown. SB43=SB431542, a TGF-βR1 antagonist. SP60=SP600125, a JNK inhibitor. U0126, a MEK1/2 (upstream of ERK1/2) inhibitor. SB20=SB203580, a p38 MAPK inhibitor. Bay=Bay11-7082, a selective NF-κB inhibitor.
