# Supplementary material for: Loss of HtrA1 serine protease induces synthetic modulation of aortic vascular smooth muscle cells
Source: PLoS One. 2018 May 16;13(5):e0196628. doi: 10.1371/journal.pone.0196628 (PMC5955505; doi:10.1371/journal.pone.0196628)
Supplement: S6 Fig — (PDF) [file pone.0196628.s006.pdf]

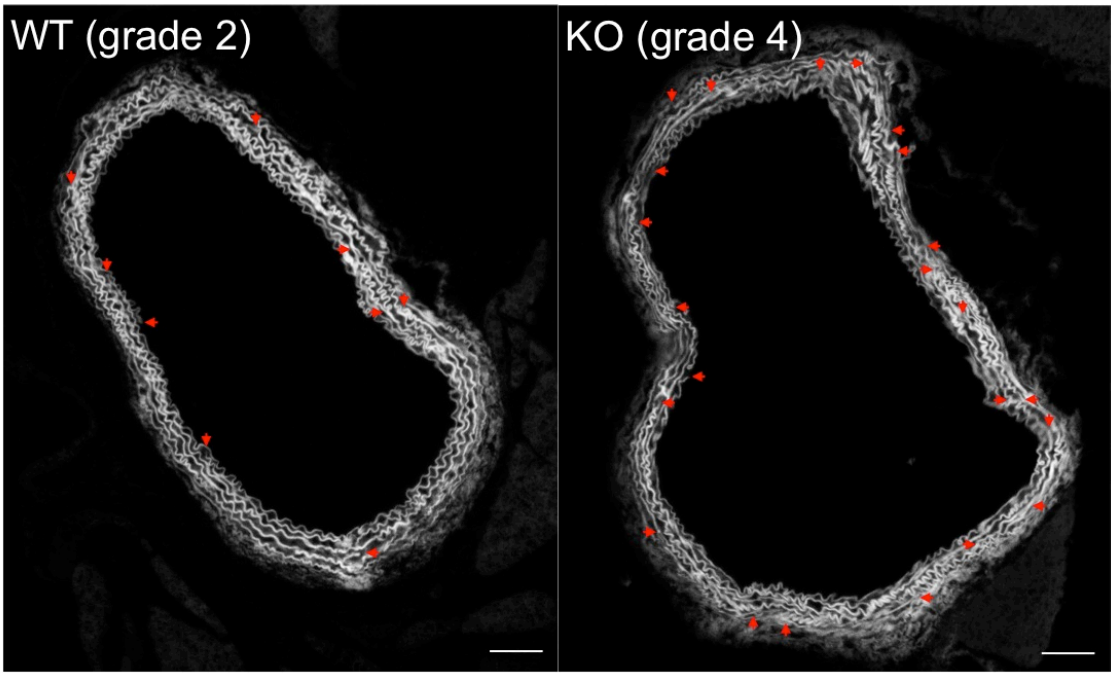

S6 Fig. Elastic fiber degradation in the aortic media of 52-wo *HtrA1*$^{-/-}$ mice. Grading of elastic fiber degradation was carried out using elastic van Gieson-stained sections based on a reported method provided in S1 File. Pictures shown are typical images of the wild type (WT) aorta (left) and the *HtrA1*$^{-/-}$ (KO) aorta (right). Red arrows indicate damaged elastic fibers. Scale bar=100 μm.
