# Supplementary material for: Loss of HtrA1 serine protease induces synthetic modulation of aortic vascular smooth muscle cells
Source: PLoS One. 2018 May 16;13(5):e0196628. doi: 10.1371/journal.pone.0196628 (PMC5955505; doi:10.1371/journal.pone.0196628)
Supplement: S7 Fig — (PDF) [file pone.0196628.s007.pdf]

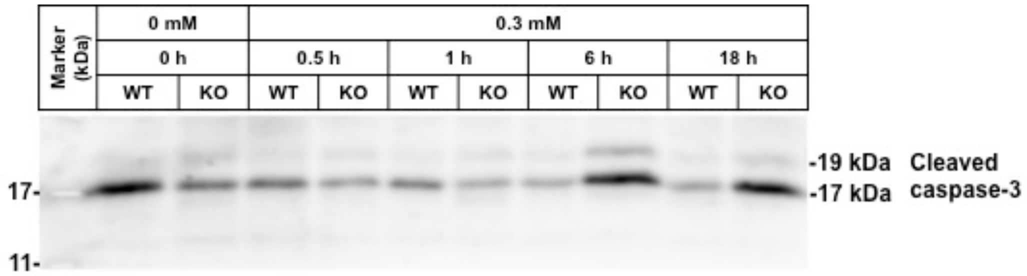

S7 Fig. Original uncropped blot of Fig 5D. Effect of H_2O_2 on the expression of cleaved caspase-3 on wild type (WT) and *HtrA1*$^{-/-}$ (KO) mouse VSMCs. WT and *HtrA1*$^{-/-}$ VSMCs were treated with 0.3 mM H_2O_2 in medium containing 0.1% FBS. Cell lysates were prepared at the time points indicated, separated by SDS- PAGE, and analyzed by Western blot for cleaved caspase-3. The anti cleaved caspase-3 antibody detected the large fragments (17/19kDa).
