# Supplementary material for: Loss of HtrA1 serine protease induces synthetic modulation of aortic vascular smooth muscle cells
Source: PLoS One. 2018 May 16;13(5):e0196628. doi: 10.1371/journal.pone.0196628 (PMC5955505; doi:10.1371/journal.pone.0196628)
Supplement: S8 Fig — (PDF) [file pone.0196628.s008.pdf]

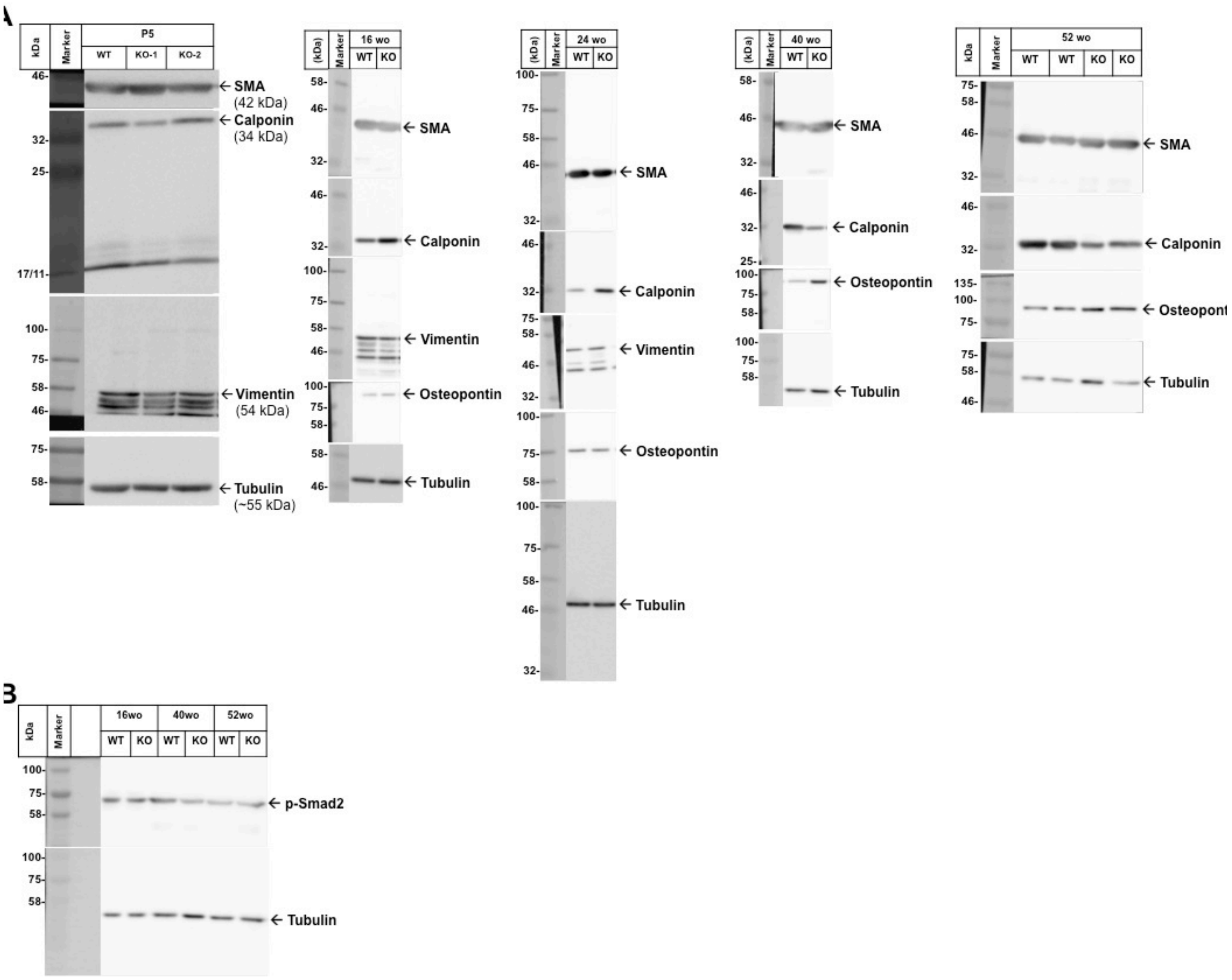

S8 Fig. Original uncropped blot of Fig 6. (A) Expression of VSMC contractile and synthetic markers in aortas of wild type (WT) and *HtrA1*$^{-/-}$ (KO) mice. **(B)** Phosphorylation of Smad2 in aortas of WT and *HtrA1*$^{-/-}$ mice. Pictures of molecular size markers (Blue Prestained Protein Standard, #P7706S, New Englang BioLabs) for each blot were taken under visible light with a Luminescent Image Analyzer LAS4000 (Fujifilm), and were imprinted to each respective blot.
