# Supplementary material for: Loss of HtrA1 serine protease induces synthetic modulation of aortic vascular smooth muscle cells
Source: PLoS One. 2018 May 16;13(5):e0196628. doi: 10.1371/journal.pone.0196628 (PMC5955505; doi:10.1371/journal.pone.0196628)
Supplement: S1 File — (PDF) [file pone.0196628.s009.pdf]

# Supplementary methods

## Grading of elastin degradation

The grading of elastic fiber degradation was carried out based on the extent of elastic fiber fragmentation and branching, according to a reported method [1] with the following modifications. One point was given to one incident of elastic fiber fragmentation or branching; the total points were summated to calculate "score" for each section from each aorta. Grade 1 = none (score 0-7); grade 2 = minimal (8-15); grade 3 = moderate (16-23); grade 4 = severe (>23) (adapted from Deckert *et al* [1]).

## PCNA immunostaining and TUNEL assay

To assess VSMC proliferation in the aorta, paraffin-embedded aorta sections were immunostained for PCNA (Biovision, 1:400). The sections were also subjected to a TUNEL assay using an in situ apoptosis detection kit (Takara) according to the manufacturer's instruction.

## Reference

1. Deckert V, Kretz B, Habbout A, Raghay K, Labbe J, Abello N, et al. Development of abdominal aortic aneurysm is decreased in mice with plasma phospholipid transfer protein deficiency. Am. J. Pathol. 2013;183:975-986.
