# Supplementary material for: Loss of HtrA1 serine protease induces synthetic modulation of aortic vascular smooth muscle cells
Source: PLoS One. 2018 May 16;13(5):e0196628. doi: 10.1371/journal.pone.0196628 (PMC5955505; doi:10.1371/journal.pone.0196628)
Supplement: S1 Table — (PDF) [file pone.0196628.s015.pdf]

S1 Table. Growth factors and inhibitors used for cell culture

Growth factor/inhibitor	Catalogue number	Manufacturer	Concentration
Recombinant murine PDGF-BB	#315-18	Peprotech	20 ng/ml
Recombinant human IGF-1	291-G1	R&D Systems	10 ng/ml
Recombinant human TGF-β1	#100-21	Peprotech	10 ng/ml
SB203580	BML-EI286	Enzo Life Sciences	10 μM
SP600125	10010466	Cayman Chemical	10 μM
SB431542	13031	Cayman Chemical	10 μM
N-acetylcysteine (NAC)	A9165	Sigma Aldrich	5 μM
Bay11-7082	10010266	Cayman Chemical	1 μM
U0126	70970	Cayman Chemical	10 μM
