# Supplementary material for: Loss of HtrA1 serine protease induces synthetic modulation of aortic vascular smooth muscle cells
Source: PLoS One. 2018 May 16;13(5):e0196628. doi: 10.1371/journal.pone.0196628 (PMC5955505; doi:10.1371/journal.pone.0196628)
Supplement: S2 Table — (PDF) [file pone.0196628.s016.pdf]

S2 Table. Forward (F) and reverse (R) primers used for qRT-PCR

Gene amplified	Primer sequences
Mouse mmp2	F, 5’-CAAGTTCCCCGGCGATGTC-3’
	R, 5’-TTCTGGTCAAGGTCACCTGT-3’
Mouse mmp9	F, 5’-GCGTCGTGATCCCCACTTAC-3’
	R, 5’-CAGGCCGAATAGGAGCGTC-3’
Mouse gapdh	F, 5’-AACATCATCCCTCATCCAC-3′
	R, 5’-CCCTGTTGCTGTAGCCGTAT-3′
