# Supplementary material for: Loss of HtrA1 serine protease induces synthetic modulation of aortic vascular smooth muscle cells
Source: PLoS One. 2018 May 16;13(5):e0196628. doi: 10.1371/journal.pone.0196628 (PMC5955505; doi:10.1371/journal.pone.0196628)
Supplement: S3 Table — (PDF) [file pone.0196628.s017.pdf]

S3 Table. Primary antibodies used for immunostaining

Antibody	Catalogue number	Reference/ Manufacturer	Dilution
Anti-HtrA1	-	[1]	1:1,000
Anti-HtrA3	-	[2]	1:1,000
Anti-smooth muscle α-actin (SMA)	ab5694	Abcam	1:100
Anti-calponin	ab46794	Abcam	1:400
Anti-vimentin	ab45939	Abcam	1:100 (for aorta) or 1:400 (for VSMCs)
Anti-osteopontin	MPIIIB101	Developmental Studies Hybridoma Bank	1:50 (for aorta) or 1:100 (for VSMCs)
Anti-cleaved caspase 3 (Asp175) (5A1E)	#9664	Cell Signaling Technology	1:400

References:

1. Oka C, Tsujimoto R, Kajikawa M, Koshiba-Takeuchi K, Ina J, Yano M, et al. HtrA1 serine protease inhibits signaling mediated by Tgfβ family proteins. Development. 2004;131:1041-1053.
2. Tocharus J, Tsuchiya A, Kajikawa M, Ueta Y, Oka C, Kawaichi M. Developmentally regulated expression of mouse HtrA3 and its role as an inhibitor of TGF-β signaling. Dev. Growth Differ. 2004;46:257–274.
