# Supplementary material for: Loss of HtrA1 serine protease induces synthetic modulation of aortic vascular smooth muscle cells
Source: PLoS One. 2018 May 16;13(5):e0196628. doi: 10.1371/journal.pone.0196628 (PMC5955505; doi:10.1371/journal.pone.0196628)
Supplement: S4 Table — (PDF) [file pone.0196628.s018.pdf]

S4 Table. Primary antibodies used for Western blotting

Antibody	Catalogue number	Reference/ Manufacturer	Dilution
Anti-HtrA1	-	[1]	1:2,000
Anti-SMA	ab5694	Abcam	1:5,000
Anti-calponin	ab46794	Abcam	1:50,000
Anti-vimentin	ab45939	Abcam	1:5,000
Anti-osteopontin	MPIIIB101	Developmental Studies Hybridoma Bank	1:1,000
Anti-p-Smad2/3 (Ser423/425)	sc-11769	Santa Cruz Biotechnology	1:2,000
Anti-cleaved caspase 3 (Asp175) (5A1E)	#9664	Cell Signaling Technology	1:1,000
Anti-α-tubulin	t9026	Sigma	1:2,000

References:

1. Oka C, Tsujimoto R, Kajikawa M, Koshiba-Takeuchi K, Ina J, Yano M, et al. HtrA1 serine protease inhibits signaling mediated by Tgfβ family proteins. Development. 2004;131:1041-1053.
